# A Straightforward Electrochemical Approach to Imine‐ and Amine‐bisphenolate Metal Complexes with Facile Control Over Metal Oxidation State

**DOI:** 10.1002/open.201600019

**Published:** 2016-04-15

**Authors:** Michael R. Chapman, Susan E. Henkelis, Nikil Kapur, Bao N. Nguyen, Charlotte E. Willans

**Affiliations:** ^1^ School of ChemistryUniversity of LeedsWoodhouse LaneLeedsLS2 9JTUnited Kingdom; ^2^ School of Mechanical EngineeringUniversity of LeedsWoodhouse LaneLeedsLS2 9JTUnited Kingdom

**Keywords:** electrochemistry, organometallic compounds, salen, Schiff bases, synthesis

## Abstract

Synthetic methods to prepare organometallic and coordination compounds such as Schiff‐base complexes are diverse, with the route chosen being dependent upon many factors such as metal–ligand combination and metal oxidation state. In this work we have shown that electrochemical methodology can be employed to synthesize a variety of metal–salen/salan complexes which comprise diverse metal–ligand combinations and oxidation states. Broad application has been demonstrated through the preparation of 34 complexes under mild and ambient conditions. Unprecedented control over metal oxidation state (M^II/III/IV^ where M=Fe, Mn) is presented by simple modification of reaction conditions. Along this route, a general protocol‐switch is described which allows access to analytically pure Fe^II/III^–salen complexes. Tuning electrochemical potential, selective metalation of a Mn/Ni alloy is also presented which exclusively delivers Mn^II/IV^–salen complexes in high yield.

## Introduction

Schiff‐base ligands are amongst the most widely studied chelators in inorganic chemistry, with salen‐type ligands (iminebisphenolates) forming a popular class of Schiff‐bases. The nitrogen atoms on the backbone may also be saturated to form salan‐type (amine‐bisphenolate) ligands. Salen‐type ligands in particular have been coordinated to a broad range of metal centers and have been used widely in catalysis, in addition to biomedical and materials applications.^1^, ^2^, ^3^, ^4^, ^5^, ^6^, ^7^, ^8^ Synthetic methods to salen complexes are relatively diverse and are very much dependent upon the desired metal and the oxidation state of the metal center. A common method to prepare metal–salens involves reaction of the ligand with metal acetate, with high temperatures often being necessary (e.g., Fe and Mn) and column chromatography being required for purification (e.g., Cu), often resulting in low yields.^9^ Other general methods include reaction of the ligand with metal alkoxide^10^ or amide precursors,^11^, ^12^ or reaction with metal halide in the presence of a base.^13^, ^14^ The use of sensitive precursors can lead to complicated product mixtures, particularly where metal–alkoxides are used due to the process being an equilibrium reaction.^3^ Metal‐salts generated when metal halides are used may coordinate to the Lewis‐basic oxygen atoms of the salen, leading to problems with purification.

**Scheme 1:**
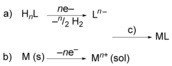
Electrochemical synthesis of metal–ligand complexes: a) reduction of HL ligand; b) oxidation of metal; c) combination of ligand and metal.

We have previously developed an electrochemical method for the preparation of Cu^I^–*N‐*heterocyclic carbene (NHC) complexes.^15^, ^16^, ^17^ The route was found to be simple, efficient, and versatile, and does not require the use of high temperatures or basic conditions. During the electrochemical reaction, an imidazolium (HL) ligand precursor is reduced at the cathode, releasing H_2_ as the only by‐product to form a free NHC (L). Concomitantly, oxidation of the sacrificial copper anode occurs, liberating Cu^+^ ions into solution. These two species combine to deliver the desired Cu^I^−NHC complex. To complement these findings and extend the current state‐of‐the‐art,^18^, ^19^, ^20^, ^21^, ^22^, ^23^, ^24^, ^25^ it was hypothesized that any HL ligand with an appropriate reduction potential could be used with a range of metals to electrochemically prepare organometallic complexes in a simple and economical manner. Herein, we report an important extension to the electrochemical syntheses through the use of different ligand types and transition metals (Scheme 1).

## Results and Discussion

**Scheme 2:**
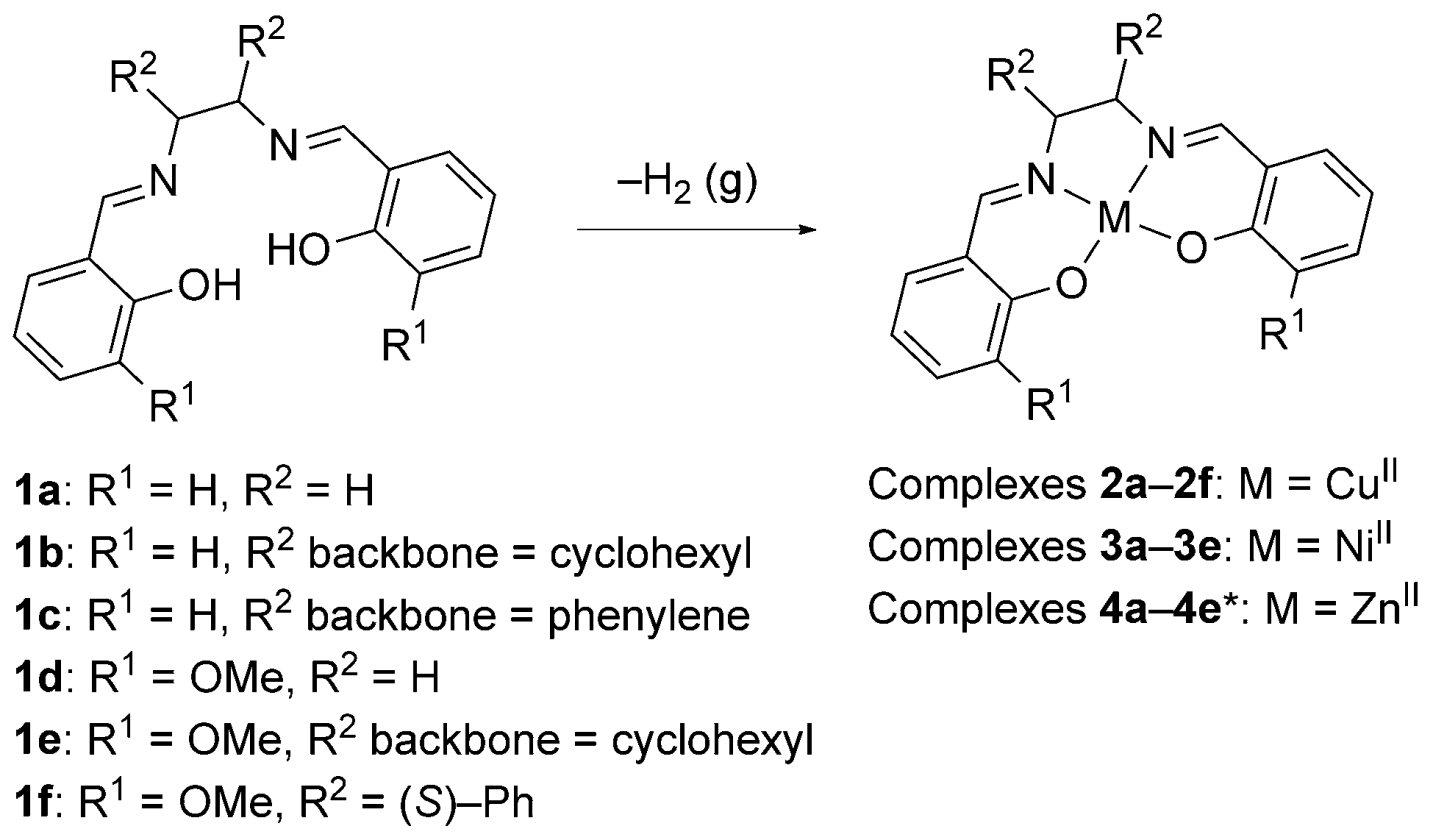
Electrochemical synthesis of metal‐salen complexes. *Reagents and conditions*: ligand precursor (1.0 mmol), Bu_4_NBF_4_ (0.03 mmol), CH_3_CN (50 mL), applied potential (22–25 V, maintaining 50 mA current), rt, 90 min. Yields available in Table 1. Zn^II^ complexes **4 a**–**4 e** contain one molecule of coordinating H_2_O from solvent.

At the outset, scope and generality of the method were evaluated; therefore, ligand precursors **1 a**–**1 h** were prepared using literature procedures and fully characterized (Scheme 2).^26^, ^27^, ^28^ Cyclic voltammetry of ligands **1 a**–**1 e** indicated reduction potentials in the range −2.04 to −2.55 V (vs. FeCp_2_/FeCp_2_^+^), similar to those found for imidazolium salts (see Supporting Information). Ligand precursors **1 a**–**1 e** were dissolved in hydrous acetonitrile, and two metal electrodes of copper, nickel, or zinc were inserted into the solution under an atmosphere of air. A potential was applied across the electrodes, maintaining a constant current of 50 mA for 90 min under aerobic conditions. The complexes precipitated from solution and were collected via vacuum filtration, to obtain analytically pure product in good yield (Scheme 2 and Table 1).

**Table 1:** Isolated yields (%) of Cu–, Ni–, and Zn–salen complexes.

Cu^II^ [Yield / %]	Ni^II^ [Yield / %]	Zn^II^ [Yield / %]
**2 a** (79)	**3 a** (91)	**4 a** (88)
**2 b** (54)	**3 b** (84)	**4 b** (79)
**2 c** (81)	**3 c** (80)	**4 c** (83)
**2 d** (86)	**3 d** (77)	**4 d** (77)
**2 e** (78)	**3 e** (88)	**4 e** (94)
**2 f** (58)		

**Figure 1:**
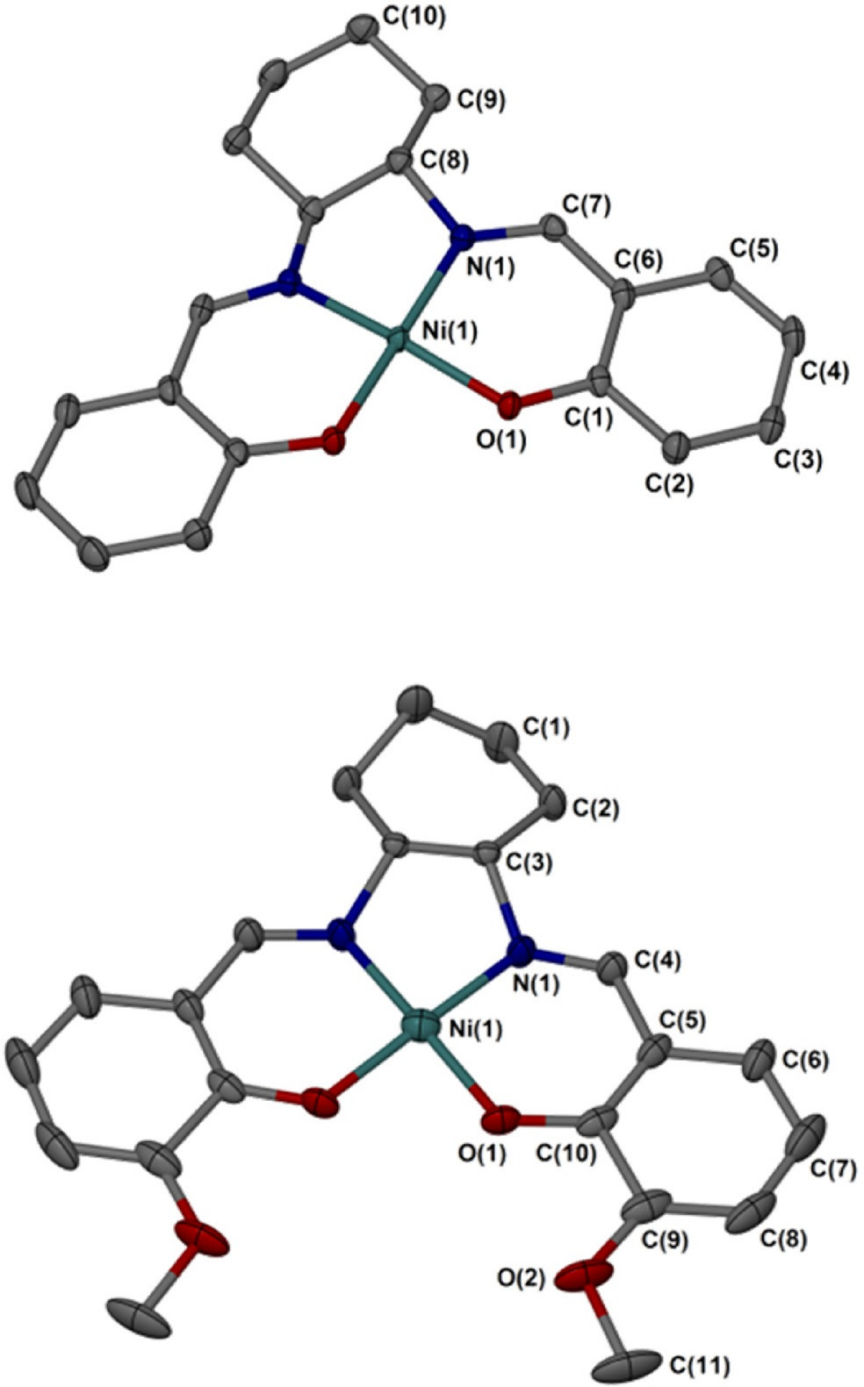
Top: molecular structure of Ni^II^–salen **3 b**. Bottom: molecular structure of Ni^II^–salen **3 e**. Ellipsoids are drawn at the 50 % probability level; hydrogen atoms are omitted for clarity.

In all cases, complexes were characterized by high‐resolution mass spectrometry (HRMS) and elemental analysis. Diamagnetic Ni^II^ and Zn^II^ complexes were also subjected to NMR spectroscopic analysis. Whilst only Cu^I^ species were observed in the electrochemical synthesis of Cu–NHCs,^15^ in the presence of salen ligands, Cu^II^ (or Ni^II^/Zn^II^) products were isolated solely. This is likely due to the presence of two reducible HL sites per ligand, in addition to the stabilization of M^2+^ complexes by salen‐type chelating ligands. Further, Ni‐complexes **3 b** and **3 e** were characterized in the solid‐state using X‐ray crystallography, with both complexes illustrating one ligand coordinating each metal through two phenoxide oxygen atoms and two imine donors, with distorted square planar environments around the metal centers (Figure 1).

**Figure 2:**
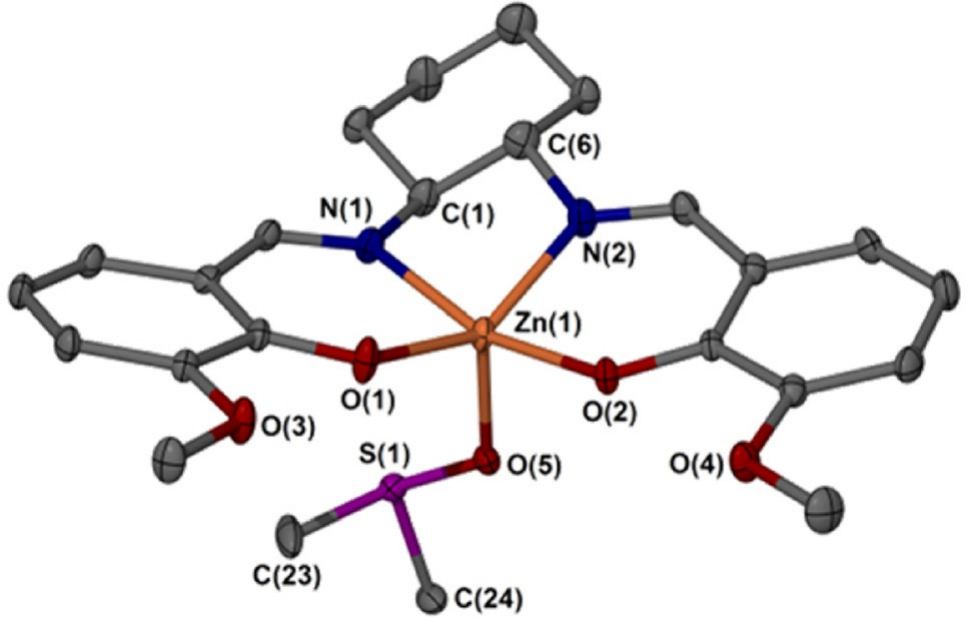
Molecular structure of Zn^II^–salen **4 e**⋅(DMSO). Ellipsoids are drawn at the 50 % probability level; hydrogen atoms are omitted for clarity.

Interestingly, during the formation of Zn complexes, one molecule of water coordinates to the metal center in each case, which is consistent with microanalytical data. The solid‐state structure of **4 e** exhibits a coordinated dimethyl sulfoxide molecule which has displaced the water during crystallization (Figure 2). In the absence of bulky *ortho*‐substituents on the phenoxide moiety, Zn^II^‐salens are known to dimerize through axial coordination of one of the oxygen atoms of each ligand to the Zn^II^ center of a neighboring unit.^29^ In the presence of other donor ligands such as water or dimethyl sulfoxide, dimerization is suppressed.

**Scheme 3:**
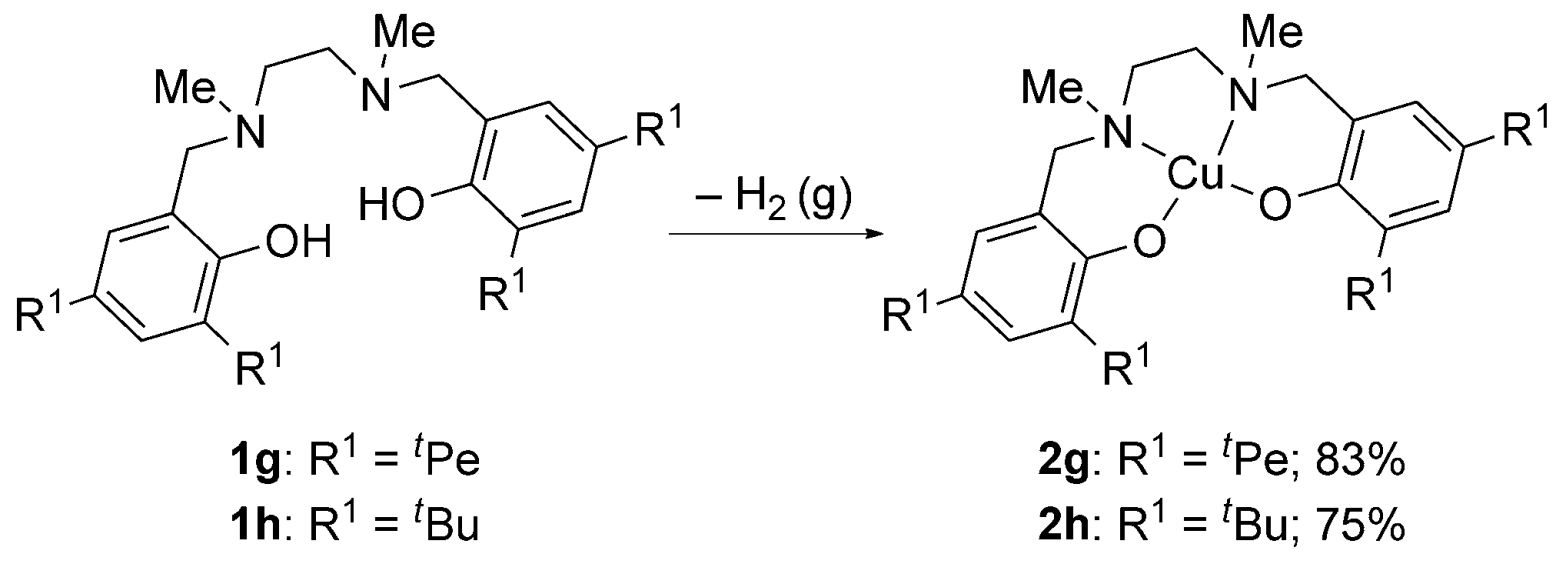
Electrochemical synthesis of Cu^II^–salan complexes with isolated yields. *Reagents and conditions*: ligand precursor (1.0 mmol), Bu_4_NBF_4_ (0.03 mmol), CH_3_CN (50 mL), applied potential (22–25 V, maintaining 50 mA current), rt, 90 min, yields shown in the scheme.

Reduction (often hydrogenation) of the salen ligand produces a new tetradentate ligand, coined as salan. Such ligands do not possess a C=N double bond, resulting in a more flexible structure around the metal center which is more resistant to hydrolysis. Several metal–salan complexes have been reported, namely with Cu,^30^, ^31^, ^32^, ^33^ Co,^31^ Ni,^33^, ^34^ V,^35^, ^36^ and Zn.^37^ However, common to their syntheses are the use of refluxing temperatures, acidic waste‐streams, and often addition of strong base to allow the products to precipitate from the reaction mixture for isolation. Therefore, N‐saturated salan precursors **1 g**–**h** underwent electrochemical conversion in a similar manner to their unsaturated analogues, using two copper plates with hydrous solvent under aerobic conditions. Following 90 min reaction time, analytically pure Cu^II^–salan complexes **2 g** and **2 h** were filtered from solution in 83 and 75 % isolated yield, respectively, with no further purification required (Scheme 3).

**Figure 3:**
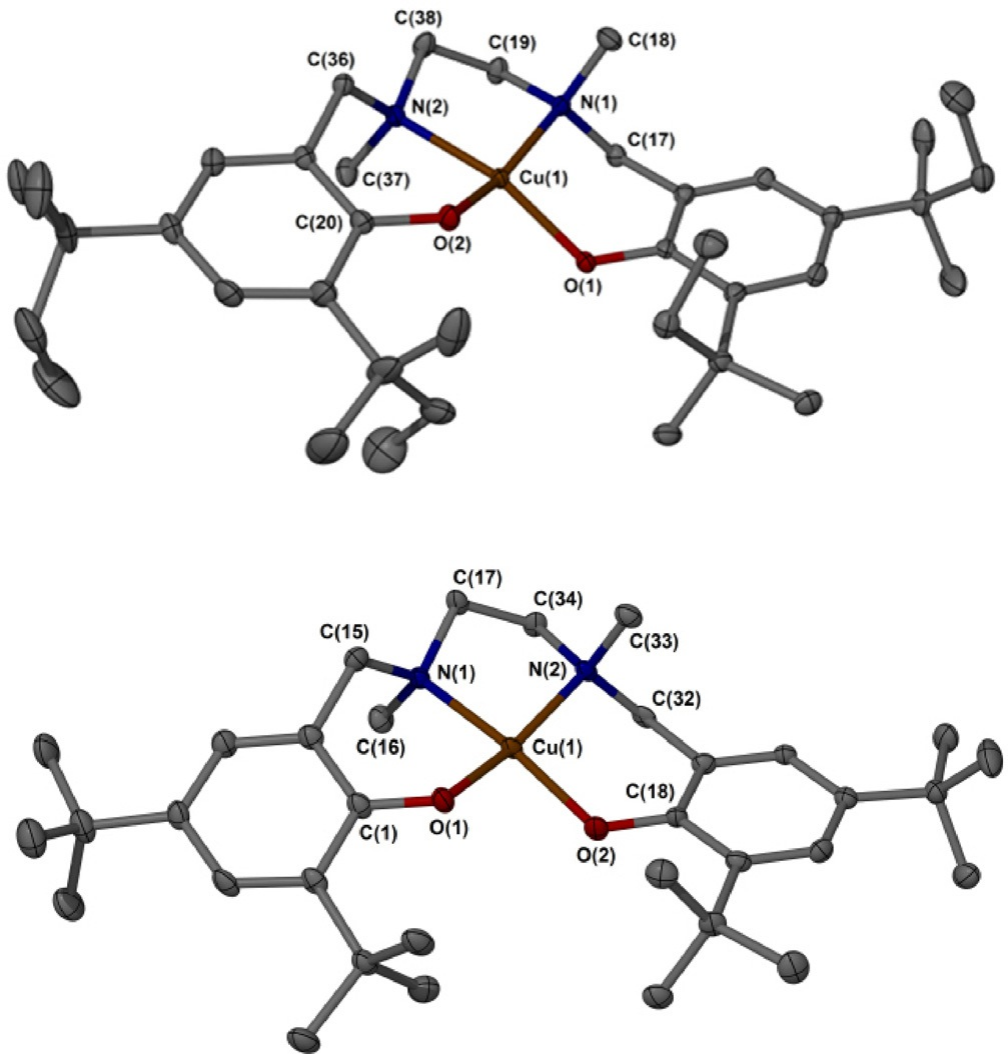
Top: molecular structure of Cu^II^–salan **2 g**. Bottom: molecular structure of Cu^II^–salan **2 h**. Ellipsoids are drawn at the 50 % probability level; hydrogen atoms are omitted for clarity.

Formation of complexes **2 g** and **2 h** was monitored via high‐resolution mass spectrometry and confirmed by microanalysis. In addition, dark green needles of each complex were isolated and analyzed by X‐ray crystallography (Figure 3).

**Scheme 4:**
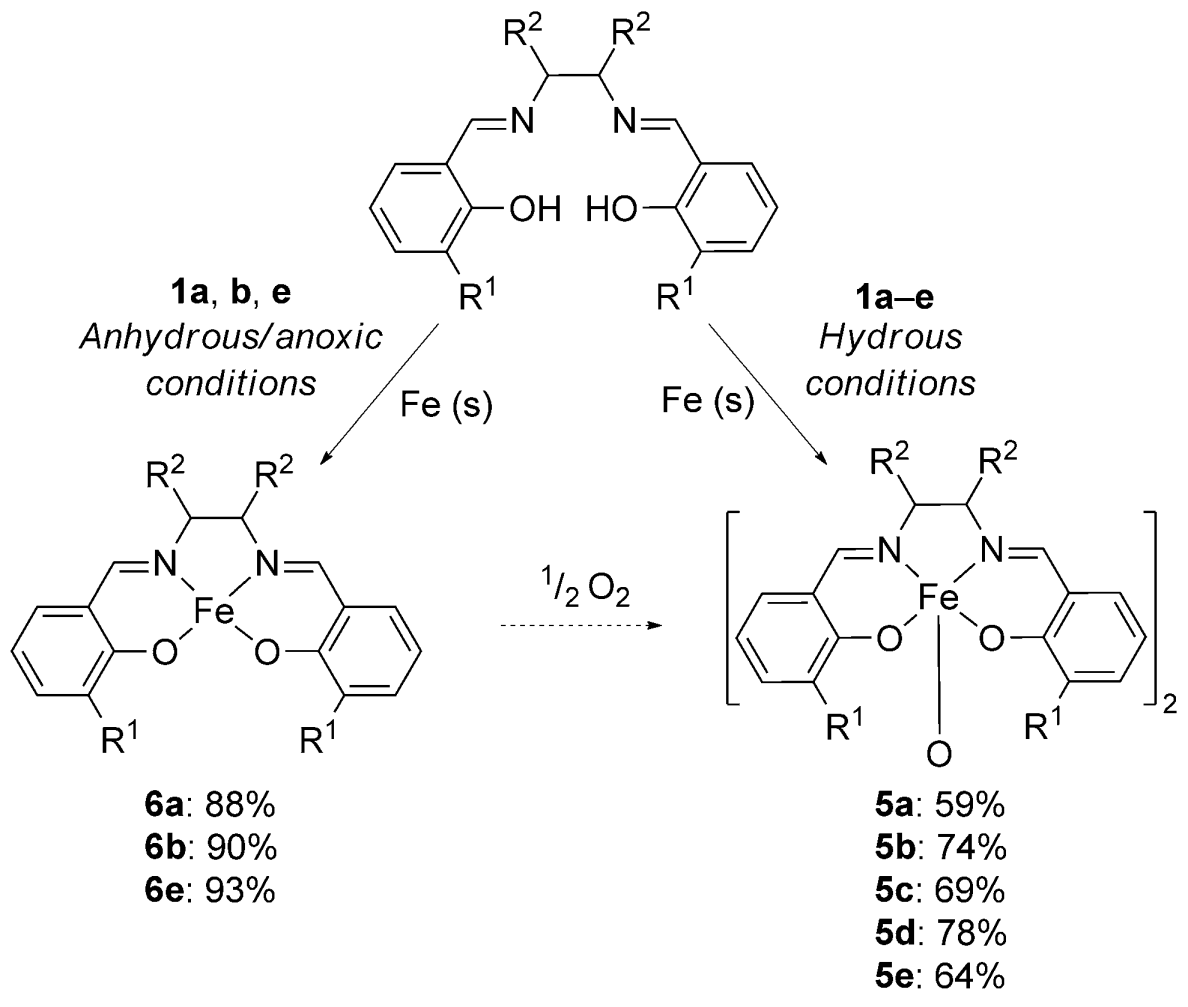
Selective electrochemical synthesis of Fe^III^–salen (right route) and Fe^II^–salen (left route) complexes with isolated yields. *Reagents and conditions*: ligand precursor (1.0 mmol), Bu_4_NBF_4_ (0.03 mmol), CH_3_CN (50 mL), applied potential (22–25 V, maintaining 50 mA current), rt, 90 min, yields shown in the scheme.

**Figure 4:**
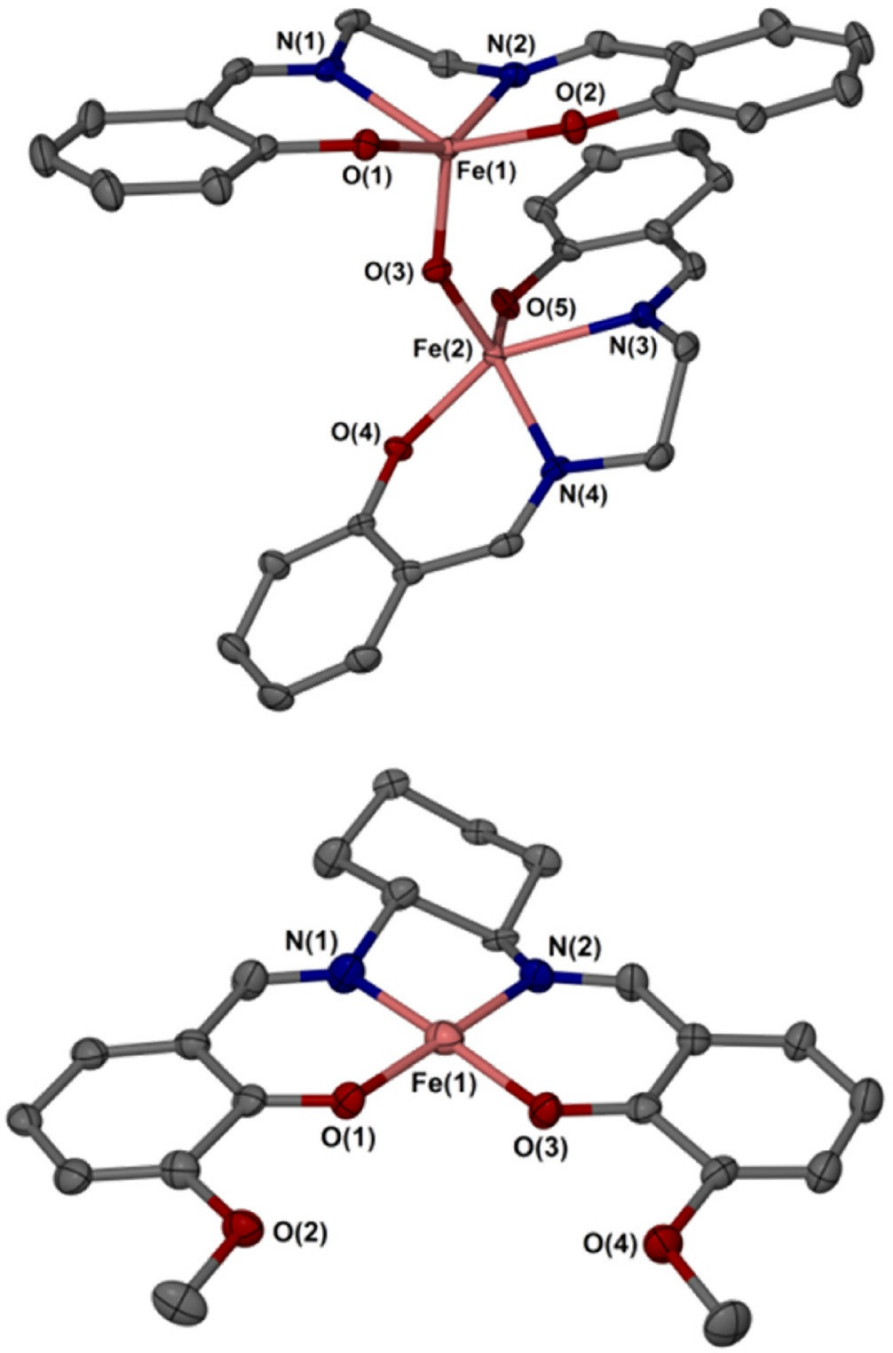
Top: molecular structure of Fe^III^–salen **5 a**, ellipsoids are drawn at the 50 % probability level. Bottom: molecular structure of Fe^II^–salen **6 e**. Ellipsoids are drawn at the 40 % probability level; hydrogen atoms are omitted for clarity.

Applying the optimized (aerobic/non‐anhydrous) procedure to ligand precursors **1 a–e** using Fe electrodes, dinuclear [Fe^III^(salen)]_2_O complexes **5 a**–**e** were isolated and fully characterized (Scheme 4, right). *μ*‐Oxo‐bridges are a common feature of Fe^III^‐salens due to the high oxygen affinity of the Fe^III^ ion,^38^ with such complexes being used in catalytic transformations,^39^ or as precursors to add another ligand. The presence of oxo‐bridges suggests that Fe^II^‐salens are initially formed electrochemically, and subsequently oxidize in the presence of O_2_ to Fe^III^. The solid‐state structure of **5 a** displays a square‐base pyramidal geometry around the metal center, coordinating two phenoxide oxygen atoms, two imine donors, and a bridging oxo group (Figure 4). All Fe^III^ complexes were characterized via high‐resolution mass spectrometry and elemental analysis.

Despite Fe^III^–salens becoming very well established within the synthetic community, isolation of Fe^II^–salen complexes is much less common due to their air‐ and moisture‐sensitivity, which presents challenges regarding their syntheses and handling. Such sensitivity largely originates from the Fe^II^ center which is forced to occupy highly distorted square‐planar geometry; the upshot of which is formation of a highly reactive metal complex. One approach toward the preparation of Fe^II^‐salens involves the organometallic reagent, Fe_2_Mes_4_, which is prepared through reaction of ferrous halide with preformed mesityl Grignard.^40^ However, Fe_2_Mes_4_ is highly air‐sensitive and difficult to handle itself, as are other common reagents such as Fe[N(SiMe_3_)_2_]_2_ which is prepared via reaction of Fe^II^ halide with Li[N(SiMe_3_)_2_], followed by high‐vacuum distillation of the highly sensitive product. Another reported synthetic method involves reaction of ligand precursors with Fe(OAc)_2_,^9^ though in our hands this route furnishes an Fe^III^ complex when ligand precursor **1 b** is used (see Supporting Information), as do other literature examples.^41^ Fe^II^–salens have been rendered inactive in catalysis in some cases, which may be explained by the presence of Fe^III^.^12^

Similar to our electrochemical methodology, Sousa and co‐workers developed an electrochemical procedure toward the synthesis of Fe^II/III^ complexes of 1‐ and 2‐substituted pyridines, with control over oxidation state for one example.^42^ Despite simple product isolation in most cases, the resulting FeL_2_ complexes were not air‐ or moisture‐sensitive, with each product forming an unreactive hydrated complex. Through using anhydrous and anoxic conditions in the electrochemical synthesis of Fe complexes, we have accessed highly air‐sensitive, spectroscopically pure Fe^II^‐salens **6 a**, **6 b**, and **6 e** in a simple and efficient manner in very high yields and relatively short reaction times (Scheme 4, left). All complexes were characterized via high‐resolution mass spectrometry and microanalysis, with single crystals of complex **6 e** also subject to X‐ray crystallographic analysis (Figure 4). Magnetic susceptibility measurements for each complex were performed in solution by means of the Evans NMR method, with effective magnetic moments calculated between 4.9–5.0 *μ_B_*, indicative of high‐spin Fe^II^ (see Supporting Information).

**Scheme 5:**
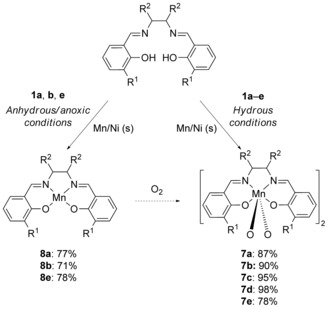
Selective electrochemical synthesis of Mn^IV^–salen (right route) and Mn^II^–salen (left route) complexes using a bimetallic Mn/Ni alloy, with isolated yields. *Reagents and conditions*: ligand precursor (1.0 mmol), Bu_4_NBF_4_ (0.03 mmol), CH_3_CN (50 mL), applied potential (22–25 V, maintaining 50 mA current), rt, 90 min, yields shown in the scheme.

**Figure 5:**
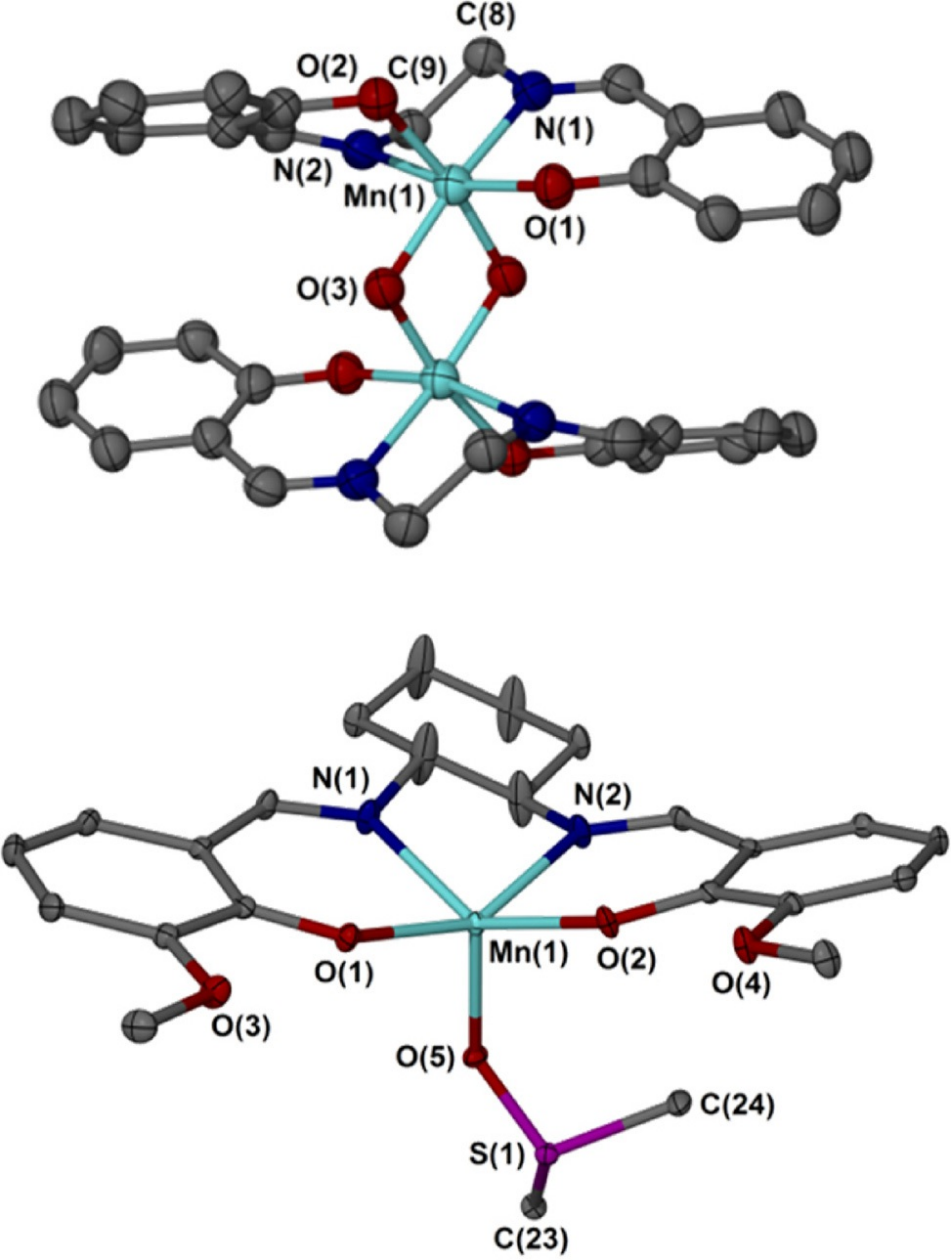
Top: molecular structure of Mn^IV^–salen **7 a**. Bottom: molecular structure of Mn^II^‐salen **8 e**⋅(DMSO). Ellipsoids are drawn at the 50 % probability level; hydrogen atoms are omitted for clarity.

Whilst Mn^IV^‐oxo complexes of Schiff bases appeared in the early 1990s, their syntheses involves reaction of the corresponding Mn(L)(acac) with H_2_O_2_ (L=Schiff base), or oxidation of a basic solution containing [Mn^III^(L)]^+^ via an over pressure of air.^43^, ^44^ Pleasingly, use of a manganese/nickel alloy anode (88 % Mn, 12 % Ni) with ligand precursors **1 a**–**e** under aerobic/non‐anhydrous conditions led to electrochemical formation of their corresponding bis(*μ*_2_‐oxo) [Mn^IV^(salen)]_2_O_2_ complexes **7 a**–**e** in high yield (Scheme 5, right). The use of this alloy enables high concentration of a brittle (non‐machinable) metal, such as Mn, to be incorporated into the electrode surface. The potential required to liberate Mn^2+^ ions into solution is 0.9 V more negative than that of Ni^2+^, allowing selective metalation of **1 a**–**e** at Mn with no corresponding Ni‐salen detected by elemental analysis, X‐ray fluorescence, or atomic absorption spectroscopy (see Supporting Information). All Mn^IV^ complexes were characterized using high‐resolution mass spectrometry and elemental analysis, with complexes **7 a** and **7 e** further probed by single crystal X‐ray diffraction analysis (Figure 5 and Supporting Information).

Akin to Fe^II^–salens, analogous complexes of Mn^II^ are typically prepared using Mn(OAc)_2_, with subsequent exposure to air providing access to their higher‐valent Mn congeners.^9^ However, the intermediate Mn^II^–salen complexes are often not isolated on account of their sensitivity, with our efforts regarding reaction of precursor **1 a** with Mn(OAc)_2_ exclusively producing an acetate‐bridged [Mn^III^(salen)(OAc)]_*n*_ polymer (see Supporting Information). In terms of electrochemical synthesis, early work by Sousa developed a protocol to salicylaldiminate complexes of Mn^III^ which are proposed to form via their neutral Mn^II^ counterparts.^45^ However, isolation of Mn^II^ Schiff base complexes which are not stabilized by additional pyridyl donors are much less common due to their high reactivity.

Employing anhydrous/anaerobic conditions to our method, analytically pure Mn^II^‐salen complexes **8 a**, **8 b** and **8 e** were accessed under equally mild and general conditions to those outlined within. All complexes were characterized by high‐resolution mass spectrometry and microanalysis, and each exhibits solution magnetic moments between 5.7–5.9 *μ_B_*, characteristic of high‐spin Mn^II^ (see Supporting Information). Single crystals of complex **8 e** were further analyzed via X‐ray crystallography, illustrating a square‐based pyramidal Mn^II^ metal center coordinated to a single salen ligand, with one molecule of solvating dimethyl sulfoxide (DMSO, crystallization solvent) also occupying the coordination sphere (Figure 5, bottom).

## Conclusion

Although metal–salen complexes may be accessed relatively easily in some cases, in other circumstances (e.g., Fe^II^‐ and Mn^II^–salens) high temperatures, sensitive precursors, and by‐product contamination can cause problems. Synthetic methods are diverse and are dependent upon the ligand, metal, and metal oxidation state. This work describes a ‘one size fits all’ approach to the synthesis of metal–salen and metal–salan complexes using an electrochemical procedure that is simple, efficient, clean, and high yielding. The electrochemical route has been shown to be versatile with regard to ligand, metal, and metal oxidation state, and could be applied to a broad array of HL‐type ligand precursors and metals. Furthermore, a bimetallic alloy can be used in the system, with remarkable control over metal selectivity through applied potential. It is envisaged that this synthetic route may be used generally by organometallic, synthetic, supramolecular, and materials scientists to easily prepare organometallic and coordination species which are otherwise difficult or impossible to access.

## Experimental Section

### General procedure for Cu^II^, Ni^II^, Zn^II^, Fe^III^, and Mn^IV^ complexes

A three‐necked round bottomed flask equipped with stirrer bar was charged with salen precursor (1.0 mmol), tetrabutylammonium tetrafluoroborate (0.03 mmol), and CH_3_CN (50 mL). Two metal electrodes (30×10×1 mm) were introduced to the solution and a potential applied via an external power supply [22.0–25.0 V] to maintain a constant current of 50.0 mA for 90 min. The resulting precipitate was collected via vacuum filtration, washed with CH_2_Cl_2_ (2×10 mL), water (2×30 mL), and diethyl ether (3×30 mL) and dried in vacuo to deliver the corresponding M–salen complex as a microcrystalline solid.

### General procedure for Fe^II^ and Mn^II^ complexes

A flame‐dried three‐necked round bottomed flask equipped with stirrer bar was charged with salen precursor (1.0 mmol) and tetrabutylammonium tetrafluoroborate (0.03 mmol) and further dried in vacuo. Anhydrous (anoxic) CH_3_CN (50 mL) was added via cannula, and the solution further degassed via bubbling a stream of argon for about 30 min through the solution. Two metal electrodes (30×10×1 mm) were introduced to the solution and a potential applied via an external power supply (22.0–25.0 V, operating in CV mode) to maintain a constant current of 50.0 mA for 90 min. The suspended precipitate was isolated via cannula filtration and washed with anhydrous (anoxic) CH_3_CN (2×15 mL), followed by rinsing with anhydrous (anoxic) diethyl ether (3×15 mL) before drying in vacuo to deliver the corresponding M–salen complex as a (highly air‐ and moisture‐sensitive) powder.

Full synthetic procedures to all ligand precursors and metal complexes, analytical data, and crystallographic data can be found in the Supporting Information. CV measurements, magnetic susceptibility measurements, and atomic adsorption and X‐ray fluorescence data are also provided in the Supporting Information, in addition to photographs to show the electrochemical reactor configuration.

## Supporting information

As a service to our authors and readers, this journal provides supporting information supplied by the authors. Such materials are peer reviewed and may be re‐organized for online delivery, but are not copy‐edited or typeset. Technical support issues arising from supporting information (other than missing files) should be addressed to the authors.

Supplementary

## References

[1] T. Katsuki , Chem. Soc. Rev. 2004, 33, 437–444. 15354225 10.1039/b304133f

[2] C. Baleizão , H. Garcia , Chem. Rev. 2006, 106, 3987–4043. 16967927 10.1021/cr050973n

[3] P. G. Cozzi , Chem. Soc. Rev. 2004, 33, 410–421. 15354222 10.1039/b307853c

[4] J. K. H. Hui , Z. Yu , M. J. MacLachlan , Angew. Chem. Int. Ed. 2007, 46, 7980–7983; 10.1002/anie.200702680 17849495

[5] A. K. Crane , M. J. MacLachlan , Eur. J. Inorg. Chem. 2012, 17–30.

[6] A. W. Kleij , Dalton Trans. 2009, 4635–4639. 19513469 10.1039/b902866h

[7] S. Medina , A. S. Henderson , J. F. Bower , M. C. Galan , Chem. Commun. 2015, 51, 8939–8941. 10.1039/c5cc02552d 25925803

[8] Y. Hitomi , Y. Iwamoto , A. Kashida , M. Kodera , Chem. Commun. 2015, 51, 8702–8704. 10.1039/c5cc02019k 25912453

[9] Y. N. Belokon , J. Fuentes , M. North , J. W. Steed , Tetrahedron 2004, 60, 3191–3204.

[10] E. L. Whitelaw , M. G. Davidson , M. D. Jones , Chem. Commun. 2011, 47, 10004–10006. 10.1039/c1cc13910j 21833423

[11] F. M. Kerton , A. C. Whitwood , C. E. Willans , Dalton Trans. 2004, 2237–2244. 15278113 10.1039/b406841f

[12] C. J. Whiteoak , R. T. M. de Rosales , A. J. P. White , G. J. P. Britovsek , Inorg. Chem. 2010, 49, 11106–11117. 21062026 10.1021/ic1016998

[13] R. R. Chowdhury , A. K. Crane , C. Fowler , P. Kwong , C. M. Kozak , Chem. Commun. 2008, 94–96. 10.1039/b713647a 18399411

[14] R. K. Dean , C. I. Fowler , K. Hasan , K. Kerman , P. Kwong , S. Trudel , D. B. Leznoff , H.-B. Kraatz , L. N. Dawe , C. M. Kozak , Dalton Trans. 2012, 41, 4806–4816. 22388465 10.1039/c2dt12242a

[15] B. R. M. Lake , E. K. Bullough , T. J. Williams , A. C. Whitwood , M. A. Little , C. E. Willans , Chem. Commun. 2012, 48, 4887–4889. 10.1039/c2cc30862b 22498755

[16] E. K. Bullough , M. A. Little , C. E. Willans , Organometallics 2013, 32, 570–577.

[17] M. R. Chapman , Y. M. Shafi , N. Kapur , B. N. Nguyen , C. E. Willans , Chem. Commun. 2015, 51, 1282–1284. 10.1039/c4cc08874c 25476754

[18] J. J. Habeeb , D. G. Tuck , F. H. Walters , J. Coord. Chem. 1978, 8, 27–33.

[19] N. Kumar , D. G. Tuck , Can. J. Chem. 1982, 60, 2579–2582.

[20] L. Bustos , J. H. Green , M. A. Khan , D. G. Tuck , Can. J. Chem. 1983, 61, 2141–2146.

[21] L. Matassa , N. Kumar , D. G. Tuck , Inorg. Chim. Acta 1985, 109, 19–21.

[22] T. A. Annan , C. Peppe , D. G. Tuck , Can. J. Chem. 1990, 68, 423–430.

[23] N. Kumar , D. G. Tuck , K. D. Watson , Can. J. Chem. 1987, 65, 740–743.

[24] J. J. Habeeb , F. F. Said , D. G. Tuck , J. Chem. Soc. Dalton Trans. 1981, 118–120.

[25] T. A. Annan , J. E. Kickham , D. G. Tuck , Can. J. Chem. 1991, 69, 251–256.

[26] D. Nartop , W. Clegg , R. W. Harrington , R. A. Henderson , C. Y. Wills , Dalton Trans. 2014, 43, 3372–3382. 24382560 10.1039/c3dt53359j

[27] T. A. Immel , M. Grützke , E. Batroff , U. Groth , T. Huhn , J. Inorg. Biochem. 2012, 106, 68–75. 22112842 10.1016/j.jinorgbio.2011.08.029

[28] D.-N. Lee , H. Kim , L. Mui , S.-W. Myung , J. Chin , H.-J. Kim , J. Org. Chem. 2009, 74, 3330–3334. 19338300 10.1021/jo900133g

[29] J. Sanmartín Matalobos , A. M. García-Deibe , M. Fondo , D. Navarro , M. R. Bermejo , Inorg. Chem. Commun. 2004, 7, 311–314.

[30] R. Klement , F. Stock , H. Elias , H. Paulus , P. Pelikan , M. Valko , M. Mazur , Polyhedron 1999, 18, 3617–3628.

[31] M. Valko , R. Klement , P. Pelikan , R. Boca , L. Dlhan , A. Bottcher , H. Elias , L. Muller , J. Phys. Chem. 1995, 99, 137–143.

[32] A. Boettcher , H. Elias , E. G. Jaeger , H. Langfelderova , M. Mazur , L. Mueller , H. Paulus , P. Pelikan , M. Rudolph , M. Valko , Inorg. Chem. 1993, 32, 4131–4138.

[33] I. Correia , A. Dornyei , T. Jakusch , F. Avacilla , T. Kiss , J. Costa Pessoa , Eur. J. Inorg. Chem. 2006, 2819–2830.

[34] A. Böttcher , H. Elias , L. Müller , H. Paulus , Angew. Chem. Int. Ed. Engl. 1992, 31, 623–625;

[35] I. Correia , J. Costa Pessoa , M. T. Duarte , R. T. Henriques , M. F. M. Piedade , L. F. Veiros , T. Jakusch , T. Kiss , A. Dornyei , M. M. C. A. Castro , C. F. G. C. Geraldes , F. Avecilla , Chem. Eur. J. 2004, 10, 2301–2317. 15112220 10.1002/chem.200305317

[36] I. Correia , J. Costa Pessoa , M. T. Duarte , M. F. M. Piedade , T. Jakusch , T. Kiss , M. M. C. A. Castro , C. F. G. C. Geraldes , F. Avecilla , Eur. J. Inorg. Chem. 2005, 732–744.

[37] I. Correia , A. Dornyei , F. Avecilla , T. Kiss , J. Costa Pessoa , Eur. J. Inorg. Chem. 2006, 656–662.

[38] M. Gerloch , E. D. McKenzie , A. D. C. Towl , Nature 1968, 220, 906–907.

[39] S. K. Edulji , S. T. Nguyen , Organometallics 2003, 22, 3374–3381.

[40] A. Klose , E. Solari , C. Floriani , A. Chiesi-Villa , C. Rizzoli , N. Re , J. Am. Chem. Soc. 1994, 116, 9123–9135.

[41] K. J. Gallagher , R. L. Webster , Chem. Commun. 2014, 50, 12109–12111. 10.1039/c4cc06526c 25168587

[42] J. Sanmartín , M. R. Bermejo , J. A. Garcia-Vazquez , J. Romero , A. Sousa , Transition Met. Chem. 1993, 18, 528–530.

[43] J. W. Gohdes , W. H. Armstrong , Inorg. Chem. 1992, 31, 368–373.

[44] E. Larson , M. S. Lah , X. Li , J. A. Bonadies , V. L. Pecoraro , Inorg. Chem. 1992, 31, 373–378.

[45] L. Luaces , M. R. Bermejo , J. A. Garcia-Vazquez , J. Romero , A. Sousa , Polyhedron 1996, 15, 3717–3724.

