# Supplementary material for: A Straightforward Electrochemical Approach to Imine‐ and Amine‐bisphenolate Metal Complexes with Facile Control Over Metal Oxidation State
Source: ChemistryOpen. 2016 Apr 15;5(4):351–6. doi: 10.1002/open.201600019 (PMC4981056; doi:10.1002/open.201600019)
Supplement: Supplementary file 1 — Supplementary [file OPEN-5-351-s001.pdf]

ChemistryOPEN

Supporting Information

© 2016 The Authors. Published by Wiley-VCH Verlag GmbH & Co. KGaA, Weinheim

A Straightforward Electrochemical Approach to Imine- and Amine-bisphenolate Metal Complexes with Facile Control Over Metal Oxidation State

Michael R. Chapman,[a] Susan E. Henkelis,[a] Nikil Kapur,[b] Bao N. Nguyen,*[a] and Charlotte E. Willans*[a]

open_201600019_sm_miscellaneous_information.pdf

Supporting Information

Table of Contents

1. General considerations
2. Synthesis of salen ligand precursors **1a-f**
3. Synthesis of Cu^{II}-salen complexes, **2a-f**
4. Synthesis of Cu^{II}-salan complexes, **2g, h**
5. Synthesis of Ni^{II}-salen complexes, **3a-e**
6. Synthesis of Zn^{II}-salen complexes, **4a-e**
7. Synthesis of Fe^{III}-salen complexes, **5a-e**
8. Synthesis of Fe^{II}-salen complexes, **6a, b, e**
9. Synthesis of Mn^{IV}-salen complexes, **7a-e**
10. Synthesis of Mn^{II}-salen complexes, **8a, b, e**
11. ^{1}H NMR spectra of Ni^{II}-salen complexes, **3a-e**
12. ^{1}H NMR spectra of Zn^{II}-salen complexes, **4a-e**
13. Cyclic voltammetry measurements
14. Atomic absorption spectroscopy
15. X-ray fluorescence analysis
16. Magnetic susceptibility measurements
17. Electrochemical reactor configuration
18. Crystallographic details
19. References

1. General considerations

Where stated, manipulations were performed under an atmosphere of dry nitrogen by means of standard Schlenk line or Glovebox techniques. Anhydrous solvents were prepared by passing the solvent over activated alumina to remove water, copper catalyst to remove oxygen and molecular sieves to remove any remaining water, *via* the Dow-Grubbs solvent system. Deuterated chloroform and acetonitrile were dried over CaH_2, cannula filtered or distilled, and then freeze-pump-thaw degassed prior to use. All other reagents and solvents were used as supplied.

^{1}H and ^{13}C NMR spectra were recorded on a Bruker DPX300 spectrometer or a Bruker AV500 spectrometer. The values of chemical shifts are given in ppm and values for coupling constants (J) in Hz. Assignment of some ^{1}H NMR spectra was aided by the use of 2D $^{1}H^{1}H$ COSY experiments and the assignment of some $^{13}C\{^{1}H\}$ NMR spectra was aided by $^{13}C\{^{1}H\}$ dept 135 experiments. Mass spectra were collected on a Bruker Daltonics (micro TOF) instrument operating in the electrospray mode. Microanalyses were performed using a Carlo Erba Elemental Analyser MOD 1106 spectrometer. Atomic absorption spectroscopy (AAS) were performed using a Perkin-Elmer Atomic Absorption Spectrometer AAnalyst 400, operating with an air-acetylene flame. X-Ray fluorescence (XRF) analysis was obtained at the University of York on a Horiba XGT7000 X-ray analytical microscope instrument using a partial vacuum.

Salan ligand precursors *N*,*N*'-*bis*(3,5-*tert*-pentylsalicyl)-1,2-ethylenedimethylamine **1g** and *N*,*N*'-*bis*(3,5-*tert*-butylsalicyl)-1,2-ethylenedimethylamine **1h** were synthesised by literature procedures[1] and all other chemicals were obtained from commercial sources and used as received. Cu foil (99.9 % purity), Fe foil (99.5 % purity), Ni foil (99.5 % purity), Zn sticks (99.9 % purity, complexometric standard) and Mn/Ni foil (Mn 88/Ni 12) were used directly without further purification. Commercially available AAS standard solutions were obtained as 1000 $mgmL^{-1}$ stock and diluted as required using ultra-pure water.

A PSD 30/3B high performance digital power supply was used in constant voltage mode (CV), with current measurements made *via* a 15XP-B Amprobe digital multimeter, at a milliampere scale.

2. Synthesis of salen ligand precursors 1a-f

Preparation of *N,N'-bis*(salicylidene)ethylenediamine, **1a**:

Salicylaldehyde (4.50 mL, 41.0 mmol) and ethylenediamine (1.40 mL, 20.5 mmol) were stirred vigorously in refluxing ethanol (50 mL) for 30 minutes. The resulting yellow solution was cooled to 0 ˚C, upon which a bright yellow precipitate formed which was collected *via* vacuum filtration, washed with cold ethanol (10 mL) followed by diethyl ether (3 × 30 mL) and dried *in vacuo* to deliver the title compound as a microcrystalline yellow solid. Yield: 3.45 g, 12.9 mmol, 63 %. ^{1}H NMR (300 MHz, $CDCl_3$): δ (ppm) 13.19 (br s, 2H, O*H*), 8.36 (s, 2H, C*H*=N), 7.32 – 7.22 (m, 4H, Ar*H*), 6.96 – 6.83 (m, 4H, Ar*H*), 3.94 (s, 4H, NCH_2CH_2N). ^{13}C{H} NMR (75 MHz, $CDCl_3$): δ (ppm) 166.7, 161.2, 132.5, 131.6, 118.8, 118.6, 117.1, 59.9. HR-MS (ESI$^+$): *m/z* 269.1274 $[C_{16}H_{17}N_2O_2]^+$, calcd. $[M + H]^+$ 269.1285. These data are in agreement with those reported in the literature.[2]

Preparation of *trans-N,N'-bis*(salicylidene)-1,2-cyclohexanediamine, **1b**:

Salicylaldehyde (4.50 mL, 41.0 mmol) and (±)-*trans*-1,2-diaminocyclohexane (2.50 mL, 20.5 mmol) were stirred vigorously in refluxing ethanol (50 mL) for 30 minutes. The resulting yellow solution was cooled to 0 ˚C, upon which a bright yellow precipitate formed which was collected *via* vacuum filtration, washed with cold ethanol (10 mL) followed by diethyl ether (3 × 30 mL) and dried *in vacuo* to deliver the title compound as a microcrystalline yellow solid. Yield: 6.52 g, 20.2 mmol, 99 %. ^{1}H NMR (300 MHz, $CDCl_3$): δ (ppm) 13.30 (br s, 2H, O*H*), 8.26 (s, 2H, C*H*=N), 7.27 – 7.21 (m, 2H, Ar*H*), 7.16 (d, *J* = 0.6 Hz, 1H, Ar*H*), 7.13 (d, *J* = 0.6 Hz, 1H, Ar*H*), 6.89 (m, 2H, Ar*H*), 6.79 (td, *J* = 15.0, 7.5, 0.6 Hz, 2H, Ar*H*), 3.36 – 3.27 (m, 2H, NC*H*), 1.97 – 1.44 (m, 8H, cy*H*). ^{13}C{H} NMR (75 MHz, $CDCl_3$): δ (ppm) 164.8, 161.1, 132.3, 131.6, 118.8, 118.7, 116.9, 72.8, 33.2, 24.3. HR-MS (ESI$^+$): *m/z* 323.1755 $[C_{20}H_{23}N_2O_2]^+$, calcd. $[M + H]^+$ 323.1754. These data are in agreement with those reported in the literature.[2]

Preparation of *N,N'-bis*(salicylidene)-1,2-phenylenediamine, **1c**:

Salicylaldehyde (4.50 mL, 41.0 mmol) and *o*-phenylenediamine (2.18 g, 20.5 mmol) were stirred vigorously in refluxing ethanol (50 mL) for 15 minutes. The resulting orange suspension was cooled

to 0 ˚C, upon which a bright orange precipitate formed which was collected *via* vacuum filtration, washed with cold ethanol (10 mL) followed by diethyl ether (3 × 30 mL) and dried *in vacuo* to deliver the title compound as a microcrystalline orange solid. Yield: 5.76 g, 18.2 mmol, 89 %. ^{1}H NMR (500 MHz, $CDCl_3$): δ (ppm) 12.91 (br s, 2H, O*H*), 8.53 (s, 2H, C*H*=N), 7.29 – 7.21 (m, 6H, Ar*H*), 7.16 – 7.11 (m, 2H, Ar*H*), 6.95 – 6.93 (m, 2H, Ar*H*), 6.81 (td, *J* = 25.0, 12.5, 1.5 Hz, 2H, Ar*H*). ^{13}C{H} NMR (75 MHz, $CDCl_3$): δ (ppm) 163.8, 161.4, 142.6, 133.4, 132.4, 127.8, 119.8, 119.3, 119.1, 117.6. HR-MS (ESI^+): *m/z* 317.1299 $[C_{20}H_{17}N_2O_2]^+$, calcd. $[M + H]^+$ 317.1285. These data are in agreement with those reported in the literature.[2]

Preparation of *N,N'-bis*(*o*-vanillidene)ethylenediamine, **1d**:

O-vanillin (3.00 g, 19.7 mmol) and ethylenediamine (0.66 mL, 9.86 mmol) were stirred vigorously in refluxing ethanol (50 mL) for 15 minutes. The resulting yellow solution was cooled to 0 ˚C, upon which a bright yellow precipitate formed which was collected *via* vacuum filtration, washed with cold ethanol (10 mL) followed by diethyl ether (3 × 30 mL) and dried *in vacuo* to deliver the title compound as a microcrystalline yellow solid. Yield: 2.90 g, 9.86 mmol, quantitative. ^{1}H NMR (500 MHz, $CDCl_3$): δ (ppm) 13.22 (br s, 2H, O*H*), 8.30 (s, 2H, C*H*=N), 6.89 (dd, *J* = 7.5, 1.5 Hz, 2H, Ar*H*), 6.83 (dd, *J* = 7.5, 1.5 Hz, 2H, Ar*H*), 6.77 – 6.74 (m, 2H, Ar*H*), 3.93 (s, 4H, NCH_2CH_2N), 3.86 (s, 6H, OCH_3). ^{13}C{H} NMR (75 MHz, $CDCl_3$): δ (ppm) 165.6, 150.4, 147.3, 122.1, 117.4, 117.0, 113.1, 58.4, 55.0. HR-MS (ESI^+): *m/z* 329.1400 $[C_{18}H_{21}N_2O_4]^+$, calcd. $[M + H]^+$ 329.1496. These data are in agreement with those reported in the literature.[3]

Preparation of *trans-N,N'-bis(o*-vanillidene)-1,2-cyclohexanediamine, **1e**:

O-vanillin (3.00 g, 19.7 mmol) and (±)-*trans*-1,2-diaminocyclohexane (1.20 mL, 9.86 mmol) were stirred vigorously in refluxing ethanol (50 mL) for 30 minutes. The resulting yellow solution was cooled to 0 ˚C, upon which a bright yellow precipitate formed which was collected *via* vacuum filtration, washed with cold ethanol (10 mL) followed by diethyl ether (3 × 30 mL) and dried *in vacuo* to deliver the title compound as a microcrystalline yellow solid. Yield: 2.79 g, 8.29 mmol, 84 %. ^{1}H NMR (300 MHz, $CDCl_3$): δ (ppm) 13.83 (br s, 2H, O*H*), 8.24 (s, 2H, C*H*=N), 6.86 (dd, *J* = 7.8, 1.8

Hz, 2H, Ar*H*), 6.79 (dd, *J* = 7.8, 1.8 Hz, 2H, Ar*H*), 6.74 – 6.69 (m, 2H, Ar*H*), 3.86 (s, 6H, OCH_3), 3.34 – 3.27 (m, 2H, NC*H*), 1.97 – 1.86 (m, 4H, cy*H*), 1.77 – 1.70 (m, 2H, cy*H*), 1.57 – 1.25 (m, 2H, cy*H*). $^{13}C\{H\}$ NMR (75 MHz, $CDCl_3$): δ (ppm) 164.9, 151.7, 148.4, 123.3, 118.5, 118.0, 114.0, 72.5, 56.1, 33.2, 24.2. HR-MS (ESI^+): *m/z* 383.1943 $[C_{22}H_{27}N_2O_4]^+$, calcd. $[M + H]^+$ 383.1965. These data are in agreement with those reported in the literature.[3]

Preparation of *N,N'-bis*(vanillidene)-1,2-diphenyl-1,2-ethylenediamine, **1f**:

O-vanillin (3.00 g, 19.7 mmol) and (1*S*,2*S*)-(-)-1,2-diphenylethylenediamine (2.09 g, 9.86 mmol) were stirred vigorously in refluxing ethanol (50 mL) for 16 hours. The resulting yellow solution was cooled to 0 ˚C, upon which a bright yellow precipitate formed which was collected *via* vacuum filtration, washed with hot ethanol (10 mL) followed by diethyl ether (3 × 30 mL) and dried *in vacuo* to deliver the title compound as a yellow solid. Yield: 1.43 g, 3.82 mmol, 39 %. ^1H NMR (300 MHz, $CDCl_3$): δ (ppm) 13.79 (br s, 2H, O*H*), 8.35 (s, 2H, C*H*=N), 7.20 – 7.12 (m, 12H, Ar*H*), 6.89 – 6.65 (m, 4H, Ar*H*), 3.88 (s, 2H, NC*H*), 2.16 (s, 6H, OCH_3). $^{13}C\{H\}$ NMR (75 MHz, $CDCl_3$): δ (ppm) 207.1, 166.1, 128.5, 128.3, 128.0, 127.8, 123.6, 118.5, 114.4, 80.5, 56.2, 31.0. HR-MS (ESI^+): *m/z* 481.2101 $[C_{30}H_{29}N_2O_4]^+$, calcd. $[M + H]^+$ 481.2122. These data are in agreement with those reported in the literature.[4]

3. Synthesis of Cu^{II}-salen complexes, 2a-f

General procedure

A three-necked round bottomed flask equipped with stirrer bar was charged with salen precursor (1.0 mmol), tetrabutylammonium tetrafluoroborate (0.03 mmol) and acetonitrile (50 mL). Two copper electrodes (30 × 10 × 1 mm) were introduced to the solution and a potential applied *via* an external power supply (22.0 – 25.0 V, operating in CV mode) to maintain a constant current of 50.0 mA for 90 minutes. The resulting precipitate was collected *via* vacuum filtration, washed with dichloromethane (2 × 10 mL), water (2 × 30 mL) diethyl ether (3 × 30 mL) and dried *in vacuo* to deliver the corresponding Cu^{II}-salen complex as a microcrystalline solid.

Preparation of Cu^{II}-salen complex, **2a**:

Salen precursor **1a** was reacted according to the general procedure (*vide supra*), affording the product as a dark green solid. Yield: 0.26 g, 0.79 mmol, 79 %. Mp decomp. >170 ˚C. HR-MS (ESI^+): *m/z* 330.0461 $[C_{16}H_{15}CuN_2O_2]^+$, calcd. $[M + H]^+$ 330.0424. Anal. calcd. (%) for $C_{16}H_{14}N_2O_2Cu.{}^1/_3CH_2Cl_2$: C 54.14, H 4.10, N 7.80; found C 54.00, H 4.10, N 7.70.

Preparation of Cu^{II}-salen complex, **2b**:

Salen precursor **1b** was reacted according to the general procedure (*vide supra*), affording the product as a purple solid. Yield: 0.21 g, 0.54 mmol, 54 %. Electrochemical efficiency: 28%. -Mp decomp. >150 ˚C. HR-MS (ESI^+): *m/z* 384.0909 $[C_{20}H_{21}CuN_2O_2]^+$, calcd. $[M + H]^+$ 384.0894. Anal. calcd. (%) for $C_{20}H_{20}N_2O_2Cu.{}^1/_3H_2O$: C 61.61, H 5.34, N 7.19; found C 61.21, H 5.20, N 7.00.

Preparation of Cu^{II}-salen complex, **2c**:

Salen precursor **1c** was reacted according to the general procedure (*vide supra*), affording the product as a dark brown solid. Yield: 0.31 g, 0.81 mmol, 81 %. Mp decomp. >180 ˚C. HR-MS (ESI^+): *m/z* 378.0427 $[C_{20}H_{15}CuN_2O_2]^+$, calcd. $[M + H]^+$ 378.0424. Anal. calcd. (%) for $C_{20}H_{14}N_2O_2Cu$: C 63.57, H 3.73, N 7.41; found C 63.40, H 3.70, N 7.30.

Preparation of Cu^{II}-salen complex, **2d**:

Salen precursor **1d** was reacted according to the general procedure (*vide supra*), affording the product as a dark green solid. Yield: 0.34 g, 0.86 mmol, 86 %. Mp decomp. >155 ˚C. HR-MS (ESI^+): *m/z* 390.0640 $[C_{18}H_{19}CuN_2O_4]^+$, calcd. $[M + H]^+$ 390.0635. Anal. calcd. (%) for $C_{18}H_{18}N_2O_4Cu.H_2O$: C 53.00, H 4.94, N 6.87; found C 52.60, H 4.90, N 6.80.

Preparation of Cu^{II}-salen complex, **2e**:

Salen precursor **1e** was reacted according to the general procedure (*vide supra*), affording the product as a dark purple solid. Yield: 0.35 g, 0.78 mmol, 78 %. Mp decomp. >150 ˚C. HR-MS (ESI^+): *m/z* 444.1109 $[C_{22}H_{25}CuN_2O_4]^+$, calcd. $[M + H]^+$ 444.1105. Anal. calcd. (%) for $C_{22}H_{24}N_2O_4Cu.H_2O$: C 57.19, H 5.67, N 6.06; found C 57.00, H 5.65, N 6.00.

Preparation of Cu^{II}-salen complex, **2f**:

Salen precursor **1f** was reacted according to the general procedure (*vide supra*), affording the product as a pale green solid. Yield: 0.31 g, 0.58 mmol, 58 %. Mp decomp. >180 ˚C. HR-MS (ESI^+): *m/z* 542.1276 $[C_{30}H_{27}CuN_2O_4]^+$, calcd. $[M + H]^+$ 542.1261. Anal. calcd. (%) for $C_{30}H_{26}N_2O_4Cu.{}^1/_3CH_2Cl_2$: C 63.90, H 4.71, N 4.91; found C 63.80, H 4.90, N 4.80.

4. Synthesis of Cu^{II}-salan complexes, 2g, h

General procedure

A three-necked round bottomed flask equipped with stirrer bar was charged with salan precursor (1.0 mmol), tetrabutylammonium tetrafluoroborate (0.03 mmol), acetonitrile (50 mL) and heated to 40 ˚C until full dissolution was achieved, upon which the solution was allowed to cool to ambient temperature. Two copper electrodes (30 × 10 × 1 mm) were introduced to the solution and a potential applied *via* an external power supply (22.0 – 25.0 V, operating in CV mode) to maintain a constant current of 50.0 mA for 90 minutes. The resulting precipitate was collected *via* vacuum filtration, washed with dichloromethane (2 × 10 mL), water (2 × 30 mL) diethyl ether (3 × 30 mL) and dried *in vacuo* to deliver the corresponding Cu^{II}-salan complex as a microcrystalline solid.

Preparation of Cu^{II}-salan complex, **2g**:

Salan precursor **1g** was reacted according to the general procedure (*vide supra*), affording the product as a dark green solid. Green needles suitable for X-ray crystallographic analysis were grown *via* slow evaporation of a concentrated CH_2Cl_2 solution of the complex. Yield: 0.53 g, 0.83 mmol, 83 %. Mp decomp. >150 ˚C. HR-MS (ESI^+): *m/z* 642.4202 $[C_{38}H_{63}CuN_2O_2]^+$, calcd. $[M + H]^+$ 642.4180. Anal. calcd. (%) for $C_{38}H_{62}N_2O_2Cu.H_2O$: C 69.10, H 9.77, N 4.24; found C 69.00, H 9.80, N 4.15.

Preparation of Cu^{II}-salen complex, **2h**:

Salan precursor **1h** was reacted according to the general procedure (*vide supra*), affording the product as a dark green solid. Green needles suitable for X-ray crystallographic analysis were grown *via* slow evaporation of a concentrated CH_2Cl_2 solution of the complex. Yield: 0.44 g, 0.75 mmol, 75 %. Mp decomp. >180 ˚C. HR-MS (ESI^+): *m/z* 586.3575 $[C_{34}H_{55}CuN_2O_2]^+$, calcd. $[M + H]^+$ 586.3554. Anal. calcd. (%) for $C_{34}H_{54}N_2O_2Cu.{}^2/_3CH_2Cl_2$: C 64.80, H 8.68, N 4.36; found C 64.40, H 8.30, N 4.10.

5. Synthesis of Ni^{II}-salen complexes, 3a-e

General procedure

A flame-dried three-necked round bottomed flask equipped with stirrer bar was charged with salen precursor (1.0 mmol), tetrabutylammonium tetrafluoroborate (0.03 mmol) and acetonitrile (50 mL) [*N.B.* product yields were much improved by use of anhydrous solvent, whilst an atmosphere of N_2 *vs* air did not affect yield]. Two nickel electrodes (30 × 10 × 1 mm) were introduced to the solution and a potential applied *via* an external power supply (22.0 – 25.0 V, operating in CV mode) to maintain a constant current of 50.0 mA for 90 minutes. The resulting precipitate was collected *via* vacuum filtration, washed with dichloromethane (2 × 10 mL) followed by diethyl ether (3 × 30 mL) to give the crude product as a hygroscopic brown/red solid. Recrystallisation of the crude product from dichloromethane/diethyl ether delivered the corresponding Ni^{II}-salen complex as a microcrystalline solid.

Preparation of Ni^{II}-salen complex, **3a**:

Salen precursor **1a** was reacted according to the general procedure (*vide supra*), affording the product as a light brown solid. Yield: 0.30 g, 0.91 mmol, 91 %. Mp decomp. >300 ˚C. ^{1}H NMR (300 MHz, CD_3CN): δ (ppm) 9.66 (s, 2H, C*H*=N), 7.50 (d, *J* = 9.0 Hz, 2H, Ar*H*), 7.35 (t, *J* = 16.8, 9.0 Hz, 2H, Ar*H*), 6.85 (t, *J* = 16.8, 9.0 Hz, 2H, Ar*H*), 6.76 (d, *J* = 9.0 Hz, 2H, Ar*H*), 3.09 (s, 4H, NCH_2CH_2N). ^{13}C{H} NMR (75 MHz, $CDCl_3$): δ (ppm) 166.5, 162.0, 132.4, 131.5, 129.9, 118.8, 118.7, 59.8. HR-MS (ESI$^+$): *m/z* 325.0483 $[C_{16}H_{15}NiN_2O_2]^+$, calcd. $[M + H]^+$ 325.0482. Anal. calcd. (%) for $C_{16}H_{14}N_2O_2Ni$: C 59.13, H 4.34, N 8.62; found C 59.10, H 4.20, N 8.40.

Preparation of Ni^{II}-salen complex, **3b**:

Salen precursor **1b** was reacted according to the general procedure (*vide supra*), affording the product as a light red solid. Pale orange needles suitable for X-ray crystallographic analysis were grown *via* the slow diffusion of Et_2O vapours into a concentrated CH_2Cl_2 solution of the complex. Yield: 0.32 g, 0.84 mmol, 84 %. Mp decomp. >300 ˚C. ^{1}H NMR (300 MHz, $CDCl_3$): δ (ppm) 7.32 (bs, 2H, C*H*=N), 7.19 (t, *J* = 12.9, 5.7 Hz, 2H, Ar*H*), 7.02 – 6.99 (m, 4H, Ar*H*), 6.51 (t, *J* = 12.9, 5.7 Hz, 2H, Ar*H*),

3.24 – 3.19 (m, 2H, NC*H*), 2.44 – 2.38 (m, 2H, cy*H*), 1.96 – 1.89 (m, 2H, cy*H*), 1.39 – 1.27 (m, 4H, cy*H*). ^{13}C{H} NMR (75 MHz, $CDCl_3$): δ (ppm) 177.3, 168.6, 139.0, 132.7, 122.6, 119.4, 110.5, 64.5, 27.7, 23.9. HR-MS (ESI$^+$): *m/z* 379.0940 $[C_{20}H_{21}NiN_2O_2]^+$, calcd. $[M + H]^+$ 379.0951. Anal. calcd. (%) for $C_{20}H_{20}N_2O_2Ni.^3/_4H_2O$: C 61.19, H 5.52, N 7.14; found C 61.20, H 5.20, N 7.00.

Preparation of Ni^{II}-salen complex, **3c**:

Salen precursor **1c** was reacted according to the general procedure (*vide supra*), affording the product as a light red solid. Yield: 0.30 g, 0.80 mmol, 80 %. Mp decomp. >300 ˚C. ^{1}H NMR (300 MHz, $CDCl_3$): δ (ppm) 8.27 (bs, 2H, C*H*=N), 7.75 – 7.71 (m, 2H, Ar*H*), 7.35 – 7.32 (m, 4H, Ar*H*), 7.20 – 7.18 (m, 4H, Ar*H*), 6.67 (t, *J* = 14.1, 7.5 Hz, 2H, Ar*H*). ^{13}C{H} NMR (75 MHz, $CDCl_3$): δ (ppm) 166.0, 161.8, 134.4, 131.2, 130.9, 126.9, 121.5, 119.4, 116.8, 112.0. HR-MS (ESI$^+$): *m/z* 373.0482 $[C_{20}H_{15}NiN_2O_2]^+$, calcd. $[M + H]^+$ 373.0482. Anal. calcd. (%) for $C_{20}H_{14}N_2O_2Ni.H_2O$: C 61.43, H 4.12, N 7.16; found C 61.20, H 3.80, N 6.95.

Preparation of Ni^{II}-salen complex, **3d**:

Salen precursor **1d** was reacted according to the general procedure (*vide supra*), affording the product as a light orange solid. Yield: 0.30 g, 0.77 mmol, 77 %. Mp decomp. >300 ˚C. ^{1}H NMR (500 MHz, $CDCl_3$): δ (ppm) 7.46 (s, 2H, C*H*=N), 6.67 – 6.65 (m, 4H, Ar*H*), 6.44 (t, *J* = 15.0, 8.0 Hz, 2H, Ar*H*), 3.79 (s, 6H, OCH_3), 3.42 (s, 4H, NCH_2CH_2N). ^{13}C{H} NMR (125 MHz, $CDCl_3$): δ (ppm) 161.6, 156.5, 151.3, 123.1, 119.5, 114.0, 112.4, 58.4, 55.3. HR-MS (ESI$^+$): *m/z* 385.0697 $[C_{18}H_{19}NiN_2O_4]^+$, calcd. $[M + H]^+$ 385.0693. Anal. calcd. (%) for $C_{18}H_{18}N_2O_4Ni$: C 56.15, H 4.71, N 7.28; found C 55.80, H 4.70, N 7.20.

Preparation of Ni^{II}-salen complex, **3e**:

Salen precursor **1e** was reacted according to the general procedure (*vide supra*), affording the product as a light brown solid. Pale brown needles suitable for X-ray crystallographic analysis were grown *via* the slow diffusion of Et_2O vapours into a concentrated CH_2Cl_2 solution of the complex. Yield: 0.39 g, 0.88 mmol, 88 %. Mp decomp. >300 ˚C. ^{1}H NMR (300 MHz, $CDCl_3$): δ (ppm) 7.12 (s, 2H, C*H*=N), 6.63 (dd, *J* = 7.5, 1.5 Hz, 2H, Ar*H*), 6.49 (dd, *J* = 7.5, 1.5 Hz, 2H, Ar*H*), 6.34 (m, 2H, Ar*H*), 3.75 (s,

6H, OC*H*$_3$), 3.20 (m, 2H, NC*H*), 2.28 (m, 2H, cy*H*), 1.83 (m, 2H, cy*H*), 1.36 – 1.18 (m, 4H, cy*H*). ^{13}C{H} NMR (125 MHz, $CDCl_3$): δ (ppm) 168.2, 155.7, 144.1, 123.3, 120.2, 118.9, 111.0, 72.5, 55.5, 33.2, 20.2. HR-MS (ESI$^+$): *m/z* 439.1168 $[C_{22}H_{25}NiN_2O_4]^+$, calcd. $[M + H]^+$ 439.1162. Anal. calcd. (%) for $C_{22}H_{24}N_2O_4Ni.H_2O$: C 57.80, H 5.73, N 6.13; found C 57.90, H 5.50, N 5.75.

6. Synthesis of Zn^{II}-salen complexes, 4a-e

General procedure

A three-necked round bottomed flask equipped with stirrer bar was charged with salen precursor (1.0 mmol), tetrabutylammonium tetrafluoroborate (0.03 mmol) and acetonitrile (50 mL). Two zinc electrodes (30 mm length, 6 mm dia.) were introduced to the solution and a potential applied *via* an external power supply (22.0 – 25.0 V, operating in CV mode) to maintain a constant current of 50.0 mA for 90 minutes. The resulting precipitate was collected *via* vacuum filtration, washed with acetonitrile (2 × 10 mL), water (2 × 30 mL) followed by diethyl ether (3 × 30 mL) and dried *in vacuo* to deliver the corresponding Zn^{II}-salen complex as a hygroscopic off-white solid.

Preparation of Zn^{II}-salen complex, **4a**:

Salen precursor **1a** was reacted according to the general procedure (*vide supra*), affording the product as a pale yellow solid. Yield: 0.29 g, 0.88 mmol, 88 %. ^{1}H NMR (500 MHz, d_6-DMSO): δ (ppm) 8.43 (s, 2H, C*H*=N), 7.15 - 7.10 (m, 4H, Ar*H*), 6.62 (d, *J* = 14.0 Hz, 2H, Ar*H*), 6.42 (t, *J* = 9.0, 14.0 Hz, 2H, Ar*H*), 3.72 (s, 4H, NCH_2CH_2N). ^{13}C{H} NMR (75 MHz, d_6-DMSO): δ (ppm) 171.0, 168.0, 134.8, 132.8, 122.7, 119.4, 112.2, 55.8. HR-MS (ESI$^+$): *m/z* 331.0418 $[C_{16}H_{15}ZnN_2O_2]^+$, calcd. $[M + H]^+$ 331.0420. Anal. calcd. (%) for $C_{16}H_{14}N_2O_2Zn$: C 57.94, H 4.25, N 8.45; found C 57.90, H 4.30, N 8.40.

Preparation of Zn^{II}-salen complex, **4b**:

Salen precursor **1b** was reacted according to the general procedure (*vide supra*), affording the product as an off-white solid. Yield: 0.30 g, 0.79 mmol, 79 %. ^{1}H NMR (500 MHz, d_6-DMSO): δ (ppm) 8.33 (s, 2H, C*H*=N), 7.23 (dd, *J* = 2.5, 12.0 Hz, 2H, Ar*H*), 7.13 (td, *J* = 2.5, 12.0, 15.6 Hz, 2H, Ar*H*), 6.63 (d, *J* = 15.6 Hz, 2H, Ar*H*), 6.42 (t, *J* = 12.0, 15.6 Hz, 2H, Ar*H*), 3.19 (m, 2H, cy*H*), 2.45 (m, 2H, cy*H*), 1.91 (m, 2H, cy*H*), 1.38 (m, 4H, cy*H*). ^{13}C{H} NMR (75 MHz, d_6-DMSO): δ (ppm) 170.9, 164.6, 135.4, 132.7, 122.6, 119.3, 112.2, 64.5, 27.7, 23.9. HR-MS (ESI$^+$): *m/z* 385.0887

$[C_{20}H_{21}ZnN_2O_2]^+$, calcd. $[M + H]^+$ 385.0889. Anal. calcd. (%) for $C_{20}H_{22}N_2O_2Zn.{}^1/_2H_2O$: C 60.85, H 5.36, N 7.10; found C 61.00, H 5.20, N 7.00.

Preparation of Zn^{II}-salen complex, **4c**:

Salen precursor **1c** was reacted according to the general procedure (*vide supra*), affording the product as a pale yellow solid. Yield: 0.35 g, 0.93 mmol, 93 %. ^1H NMR (500 MHz, d_6-DMSO): δ (ppm) 9.01 (s, 2H, C*H*=N), 7.91 – 7.89 (m, 2H, Ar*H*), 7.42 – 7.38 (m, 4H, Ar*H*), 7.25 (td, *J* = 1.5, 6.5, 15.0 Hz, 2H, Ar*H*), 6.72 (d, *J* = 15.0 Hz, 2H, Ar*H*), 6.51 (t, *J* = 6.5, 15.0 Hz, 2H, Ar*H*). $^{13}C\{H\}$ NMR (75 MHz, d_6-DMSO): δ (ppm) 172.2, 162.8, 139.3, 136.2, 134.3, 127.2, 123.1, 119.4, 116.4, 112.9. HR-MS (ESI^+): *m/z* 379.0420 $[C_{20}H_{15}ZnN_2O_2]^+$, calcd. $[M + H]^+$ 379.0417. Anal. calcd. (%) for $C_{20}H_{17}N_2O_3Zn$: C 60.30, H 4.05, N 7.04; found C 59.90, H 4.10, N 6.80.

Preparation of Zn^{II}-salen complex, **4d**:

Salen precursor **1d** was reacted according to the general procedure (*vide supra*), affording the product as a colourless solid. Yield: 0.30 g, 0.77 mmol, 77 %. ^1H NMR (500 MHz, d_6-DMSO): δ (ppm) 8.42 (s, 2H, C*H*=N), 6.78 – 6.76 (m, 4H, Ar*H*), 6.36 – 6.33 (m, 2H, Ar*H*), 3.72 (s, 6H, OCH_3), 3.71 (s, 4H, NCH_2CH_2N). $^{13}C\{H\}$ NMR (75 MHz, d_6-DMSO): δ (ppm) 168.0, 162.1, 152.5, 126.1, 118.5, 113.0, 110.9, 56.0, 55.0. HR-MS (ESI^+): *m/z* 469.0772 $[C_{18}H_{19}ZnN_2O_4 + DMSO]^+$, calcd. $[M + H]^+$ 469.0770. Anal. calcd. (%) for $C_{18}H_{20}N_2O_5Zn$: C 52.76, H 4.92, N 6.84; found C 52.40, H 4.90, N 6.70.

Preparation of Zn^{II}-salen complex, **4e**:

Salen precursor **1e** was reacted according to the general procedure (*vide supra*), affording the product as an off-white solid. Colourless blocks suitable for X-ray crystallographic analysis were grown upon standing of a concentrated DMSO solution of the complex. Yield: 0.42 g, 0.94 mmol, 94 %. ^1H NMR (500 MHz, d_6-DMSO): δ (ppm) 8.32 (s, 2H, C*H*=N), 6.84 (d, *J* = 8.0 Hz, 2H, Ar*H*), 7.78 (d, *J* = 8.0 Hz, 2H, Ar*H*), 6.34 (t, *J* = 8.0, 15.0 Hz, 2H, Ar*H*), 3.72 (s, 6H, OCH_3), 3.20 – 3.18 (m, 2H, cy*H*), 2.45 – 2.43 (m, 2H, cy*H*), 1.91 – 1.90 (m, 2H, cy*H*), 1.43 – 1.30 (m, 4H, cy*H*). $^{13}C\{H\}$ NMR (75 MHz, d_6-DMSO): δ (ppm) 164.7, 151.9, 126.5, 118.2, 114.8, 111.4, 64.9, 63.9, 27.8, 23.9, 14.6. HR-MS

(ESI$^+$): *m/z* 445.1109 [$C_{22}H_{25}ZnN_2O_4$]$^+$, calcd. [M + H]$^+$ 445.1100. Anal. calcd. (%) for $C_{22}H_{26}N_2O_5Zn.^1/_4H_2O$: C 56.42, H 5.70, N 5.98; found C 56.20, H 5.90, N 5.60.

7. Synthesis of Fe^{III}-salen complexes, 5a-e

General procedure

A three-necked round bottomed flask equipped with stirrer bar was charged with salen precursor (1.0 mmol), tetrabutylammonium tetrafluoroborate (0.03 mmol) and acetonitrile (50 mL). Two iron electrodes (30 × 10 × 1 mm) were introduced to the solution and a potential applied *via* an external power supply (22.0 – 25.0 V, operating in CV mode) to maintain a constant current of 50.0 mA for 90 minutes. The resulting precipitate was collected *via* vacuum filtration, washed with acetonitrile (2 × 10 mL), water (2 × 30 mL), diethyl ether (3 × 30 mL) and dried *in vacuo* to deliver the corresponding Fe^{III}-salen complex as a microcrystalline solid.

Preparation of Fe^{III}-salen complex, **5a**:

Salen precursor **1a** was reacted according to the general procedure (*vide supra*), affording the product as a dark red solid. Dark red needles suitable for X-ray crystallographic analysis were grown *via* the slow diffusion of Et_2O vapours into a concentrated CH_2Cl_2 solution of the complex. Yield: 0.20 g, 0.30 mmol, 59 %. Mp decomp. >180 ˚C. HR-MS (ESI^+): *m/z* 661.0828 $[C_{32}H_{28}Fe_2N_4O_5]^+$, calcd. $[M + H]^+$ 661.0831. Anal. calcd. (%) for $C_{32}H_{29}N_4O_5Fe_2$: C 56.83, H 4.17, N 8.28; found C 56.80, H 4.20, N 8.10.

Preparation of Fe^{III}-salen complex, **5b**:

Salen precursor **1b** was reacted according to the general procedure (*vide supra*), affording the product as a red solid. Yield: 0.28 g, 0.37 mmol, 74 %. Mp decomp. >200 ˚C. HR-MS (ESI^+): *m/z* 769.1777 $[C_{40}H_{41}Fe_2N_4O_5]^+$, calcd. $[M + H]^+$ 769.1770. Anal. calcd. (%) for $C_{40}H_{40}N_4O_5Fe_2.{}^1/_4H_2O$: C 62.15, H 5.28, N 7.25; found C 61.85, H 5.20, N 7.20.

Preparation of Fe^{III}-salen complex, **5c**:

Salen precursor **1c** was reacted according to the general procedure (*vide supra*), affording the product as a dark red/brown solid. Yield: 0.26 g, 0.35 mmol, 69 %. Mp decomp. >200 ˚C. HR-MS (ESI^+): *m/z*

757.1090 [$C_{40}H_{29}Fe_2N_4O_5$]$^+$, calcd. [M + H]$^+$ 757.0831. Anal. calcd. (%) for $C_{40}H_{28}N_4O_5Fe_2.H_2O$: C 59.43, H 3.99, N 6.93; found C 59.30, H 3.80, N 6.60.

Preparation of Fe^{III}-salen complex, **5d**:

Salen precursor **1d** was reacted according to the general procedure (*vide supra*), affording the product as a red/brown solid. Yield: 0.30 g, 0.39 mmol, 78 %. Mp decomp. >180 ˚C. HR-MS (ESI$^+$): *m/z* 781.1888 [$C_{36}H_{37}Fe_2N_4O_9$]$^+$, calcd. [M + H]$^+$ 781.1254. Anal. calcd. (%) for $C_{36}H_{36}N_4O_9Fe_2.1^1/_2H_2O$: C 50.84, H 4.98, N 6.59; found C 50.80, H 4.90, N 6.50.

Preparation of Fe^{III}-salen complex, **5e**:

Salen precursor **1e** was reacted according to the general procedure (*vide supra*), affording the product as a dark brown solid. Yield: 0.28 g, 0.32 mmol, 64 %. Mp decomp. >200 ˚C. HR-MS (ESI$^+$): *m/z* 889.2180 [$C_{44}H_{49}Fe_2N_4O_9$]$^+$, calcd. [M + H]$^+$ 889.2193. Anal. calcd. (%) for $C_{44}H_{48}N_4O_9Fe_2$: C 61.54, H 5.14, N 7.14; found C 61.85, H 5.20, N 7.20.

Preparation of Fe^{III}-salen complex, **5**, from $Fe(OAc)_2$:[5a,b]

Salen precursor **1b** (0.20 g, 0.62 mmol) and anhydrous $Fe(OAc)_2$ (0.11 g, 0.62 mmol) were charged to a flame-dried Schlenk flask and stirred in anhydrous ethanol (10 mL) at 75 ˚C for 1 hour under an inert atmosphere. Upon slow cooling to room temperature, a dark purple microcrystalline solid precipitated from solution, which was collected *via* filtration under N_2 and washed with anhydrous hexane (2 × 15 mL). The solid was further purified by suspension in refluxing ethanol under N_2, followed by filtration and washing with additional hexane (3 × 20 mL) to give the Fe^{III}(salen)(OAc) complex **5** as a microcrystalline dark purple solid. Anal. calcd. (%) for $C_{22}H_{23}N_2O_4Fe$: C 60.71, H 5.33, N 6.64; found C 60.80, H 5.15, N 7.00.

8. Synthesis of Fe^{II}-salen complexes, 6a, b, e

General procedure

A flame-dried three-necked round bottomed flask equipped with stirrer bar was charged with salen precursor (1.0 mmol), tetrabutylammonium tetrafluoroborate (0.03 mmol) and further dried *in vacuo*. Anhydrous (anoxic) acetonitrile (50 mL) was added *via* cannula and the solution further degassed *via* bubbling a stream of N_2 or Ar for *ca*. 30 minutes through the solution. Two iron electrodes (30 × 10 × 1 mm) were introduced to the solution and a potential applied *via* an external power supply (22.0 – 25.0 V, operating in CV mode) to maintain a constant current of 50.0 mA for 90 minutes. The suspended violet precipitate was isolated *via* cannula filtration and washed with anhydrous (anoxic) acetonitrile (2 × 15 mL), followed by rinsing with anhydrous (anoxic) diethyl ether (3 × 15 mL) before drying *in vacuo* to deliver the corresponding Fe^{II}-salen complex as a (highly air- and moisture-sensitive) light violet powder [*N. B.* upon exposure to an atmosphere of air, the products rapidly become an orange powder, which is likely due to oxidation of the Fe centre].

Preparation of Fe^{II}-salen complex, **6a**:

Salen precursor **1a** was reacted according to the general procedure (*vide supra*), affording the product as a light violet solid. Yield: 0.28 g, 0.88 mmol, 88 %. μ_{eff} = 5.0 μ_B. HR-MS (ESI$^+$): *m/z* 323.1761 $[C_{16}H_{15}FeN_2O_2]^+$, calcd. $[M + H]^+$ 323.0477. Anal. calcd. (%) for $C_{16}H_{14}N_2O_2Fe$: C 59.66, H 4.38, N 8.70; found C 59.90, H 4.30, N 8.60.

Preparation of Fe^{II}-salen complex, **6b**:

Salen precursor **1b** was reacted according to the general procedure (*vide supra*), affording the product as a violet solid. Yield: 0.34 g, 0.90 mmol, 90 %. μ_{eff} = 4.9 μ_B. HR-MS (ESI$^+$): *m/z* 376.0870 $[C_{20}H_{21}FeN_2O_2]^+$, calcd. $[M + H]^+$ 376.0874. Anal. calcd. (%) for $C_{16}H_{14}N_2O_2Fe.{}^3/_4H_2O$: C 61.63, H 5.56, N 7.19; found C 61.50, H 5.20, N 7.10.

Preparation of Fe^{II}-salen complex, **6e**:

Salen precursor **1e** was reacted according to the general procedure (*vide supra*), affording the product as a light violet solid. Violet blocks suitable for X-ray crystallographic analysis were grown *via* slow diffusion of Et_2O vapours into a weak CH_2Cl_2 solution of the complex. Yield: 0.41 g, 0.93 mmol, 93 %. μ_{eff} = 4.9 μ_B. HR-MS (ESI^+): *m/z* 437.1160 $[C_{22}H_{25}FeN_2O_4]^+$, calcd. $[M + H]^+$ 437.1158. Anal. calcd. (%) for $C_{22}H_{24}N_2O_4Fe.{}^3/_4CH_3CN$: C 60.43, H 5.66, N 8.25; found C 60.50, H 5.40, N 8.30.

9. Synthesis of Mn^{IV}-salen complexes, 7a-e

General procedure

A three-necked round bottomed flask equipped with stirrer bar was charged with salen precursor (1.0 mmol), tetrabutylammonium tetrafluoroborate (0.03 mmol) and acetonitrile (50 mL). Two manganese/nickel (80:20 weight %) electrodes (25 × 10 × 0.25 mm) were introduced to the solution and a potential applied *via* an external power supply (22.0 – 25.0 V, operating in CV mode) to maintain a constant current of 20.0 mA for 90 minutes. The resulting precipitate was collected *via* vacuum filtration, washed with acetonitrile (2 × 10 mL) followed by diethyl ether (3 × 30 mL) and dried *in vacuo* to deliver the corresponding Mn^{IV}-salen complex as a microcrystalline solid.

Preparation of Mn^{IV}-salen complex, **7a**:

Salen precursor **1a** was reacted according to the general procedure (*vide supra*), affording the product as a brown powder. Pale brown blocks suitable for X-ray crystallographic analysis were grown *via* either (i) slow diffusion of Et_2O vapours into a concentrated binary CH_2Cl_2/DMSO solution of the complex, or (ii) standing of a weak MeCN solution of the complex to give two different solvent-dependent solid-state structures of **7a** (see section 18, Crystallographic details). Yield: 0.30 g, 0.44 mmol, 87 %. Mp decomp. >300 ˚C. HR-MS (ESI^+): *m/z* 675.0845 $[C_{32}H_{29}Mn_2N_4O_6]^+$, calcd. $[M + H]^+$ 675.0843. Anal. calcd. (%) for $C_{32}H_{28}N_4O_6Mn_2$: C 57.99, H 4.18, N 8.31; found C 58.10, H 4.25, N 8.40.

Preparation of Mn^{IV}-salen complex, **7b**:

Salen precursor **1b** was reacted according to the general procedure (*vide supra*), affording the product as a brown powder. Yield: 0.39 g, 0.45 mmol, 90 %. Mp decomp. >300 ˚C. HR-MS (ESI^+): *m/z* 783.1780 $[C_{40}H_{41}Mn_2N_4O_6]^+$, calcd. $[M + H]^+$ 783.1782. Anal. calcd. (%) for $C_{40}H_{40}N_4O_6Mn_2$: C 61.39, H 5.15, N 7.16; found C 61.20, H 5.20, N 7.20.

Preparation of Mn^{IV}-salen complex, **7c**:

Salen precursor **1c** was reacted according to the general procedure (*vide supra*), affording the product as a brown powder. Yield: 0.38 g, 0.49 mmol, 95 %. Mp decomp. >300 ˚C. HR-MS (ESI$^+$): *m/z* 771.0840 $[C_{40}H_{29}Mn_2N_4O_6]^+$, calcd. $[M + H]^+$ 771.0843. Anal. calcd. (%) for $C_{40}H_{28}N_4O_6Mn_2$: C 62.35, H 3.66, N 7.27; found C 62.30, H 3.99, N 7.00.

Preparation of Mn^{IV}-salen complex, **7d**:

Salen precursor **1d** was reacted according to the general procedure (*vide supra*), affording the product as a brown powder. Yield: 0.39 g, 0.49 mmol, 98 %. Mp decomp. >300 ˚C. HR-MS (ESI$^+$): *m/z* 795.1271 $[C_{36}H_{37}Mn_2N_4O_{10}]^+$, calcd. $[M + H]^+$ 795.1265. Anal. calcd. (%) for $C_{36}H_{36}N_4O_{10}Mn_2.H_2O$: C 53.21, H 4.71, N 6.89; found C 53.30, H 4.61, N 6.95.

Preparation of Mn^{IV}-salen complex, **7e**:

Salen precursor **1e** was reacted according to the general procedure (*vide supra*), affording the product as a brown powder. Pale brown blocks suitable for X-ray crystallographic analysis were grown *via* slow diffusion of Et_2O vapours into a concentrated binary CH_2Cl_2/DMSO solution of the complex. Yield: 0.35 g, 0.39 mmol, 78 %. Mp decomp. >300 ˚C. HR-MS (ESI$^+$): *m/z* 903.2225 $[C_{44}H_{49}Mn_2N_4O_{10}]^+$, calcd. $[M + H]^+$ 903.2204. Anal. calcd. (%) for $C_{44}H_{48}N_4O_{10}Mn_2.{}^1/_2H_2O$: C 57.96, H 5.42, N 6.14; found C 57.65, H 5.20, N 6.10.

Preparation of Mn^{III}-salen polymer, **7**, from $Mn(OAc)_2$:[5a]

Salen precursor **1a** (0.17 g, 0.62 mmol) and anhydrous $Mn(OAc)_2$ (0.11 g, 0.62 mmol) were charged to a flame-dried Schlenk flask and stirred in anhydrous ethanol (10 mL) at 75 ˚C for 1 hour under an inert atmosphere. Upon slow cooling to room temperature, a dark brown microcrystalline solid precipitated from solution, which was collected *via* filtration under N_2 and washed with anhydrous hexane (2 × 15 mL). The solid was further purified by suspension in refluxing ethanol under N_2, followed by filtration and washing with additional hexane (3 × 20 mL) to give the $[Mn^{III}(salen)(OAc)]$

polymer **7** as a microcrystalline dark brown solid. Anal. calcd. (%) for $C_{18}H_{17}N_2O_4Mn$: C 56.85, H 4.51, N 7.37; found C 56.50, H 4.50, N 7.20.

10. Synthesis of Mn^{II}-salen complexes, 8a, b, e

A flame-dried three-necked round bottomed flask equipped with stirrer bar was charged with salen precursor (1.0 mmol), tetrabutylammonium tetrafluoroborate (0.03 mmol) and further dried *in vacuo*. Anhydrous (anoxic) acetonitrile (50 mL) was added *via* cannula and the solution further degassed *via* bubbling a stream of N_2 or Ar for *ca*. 30 minutes through the solution. Two manganese/nickel (80:20 weight %) electrodes (25 × 10 × 0.25 mm) were introduced to the solution and a potential applied *via* an external power supply (22.0 – 25.0 V, operating in CV mode) to maintain a constant current of 20.0 mA for 90 minutes. The suspended pale orange precipitate was isolated *via* cannula filtration and washed with anhydrous (anoxic) acetonitrile (2 × 15 mL), followed by rinsing with anhydrous (anoxic) diethyl ether (3 × 15 mL) before drying *in vacuo* to deliver the corresponding Mn^{II}-salen complex as a (highly air- and moisture-sensitive) pale orange powder.

Preparation of Mn^{II}-salen complex, **8a**:

Salen precursor **1a** was reacted according to the general procedure (*vide supra*), affording the product as a pale orange microcrystalline powder. Yield: 0.25 g, 0.77 mmol, 77 %. μ_{eff} = 5.9 μ_B. HR-MS (ESI^+): *m/z* 321.0460 $[C_{16}H_{14}MnN_2O_2]^+$, calcd. $[M]^+$ 321.0436. Anal. calcd. (%) for $C_{16}H_{14}N_2O_2Mn$: C 59.82, H 4.39, N 8.72; found C 60.20, H 4.10, N 8.75.

Preparation of Mn^{II}-salen complex, **8b**:

Salen precursor **1b** was reacted according to the general procedure (*vide supra*), affording the product as a pale orange microcrystalline powder. Yield: 0.27 g, 0.71 mmol, 71 %. μ_{eff} = 5.7 μ_B. HR-MS (ESI^+): *m/z* 375.0903 $[C_{20}H_{20}MnN_2O_2]^+$, calcd. $[M]^+$ 375.0905. Anal. calcd. (%) for $C_{20}H_{20}N_2O_2Mn$: C 64.00, H 5.37, N 7.46; found C 64.22, H 5.00, N 7.75.

Preparation of Mn^{II}-salen complex, **8e**:

Salen precursor **1e** was reacted according to the general procedure (*vide supra*), affording the product as a pale orange microcrystalline powder. Bright yellow blocks suitable for X-ray crystallographic analysis were grown *via* slow diffusion of Et_2O vapours into a concentrated binary CH_2Cl_2/DMSO

solution of the complex. Yield: 0.33 g, 0.78 mmol, 78 %. μ_{eff} = 5.8 μ_B. HR-MS (ESI$^+$): *m/z* 429.0647 $[C_{22}H_{18}MnN_2O_4]^+$, calcd. $[M]^+$ 429.0647. Anal. calcd. (%) for $C_{22}H_{24}N_2O_4Mn$: C 60.69, H 5.56, N 6.43; found C 60.40, H 5.10, N 6.80.

11. ^{1}H NMR spectra of Ni^{II}-salen complexes, 3a-e

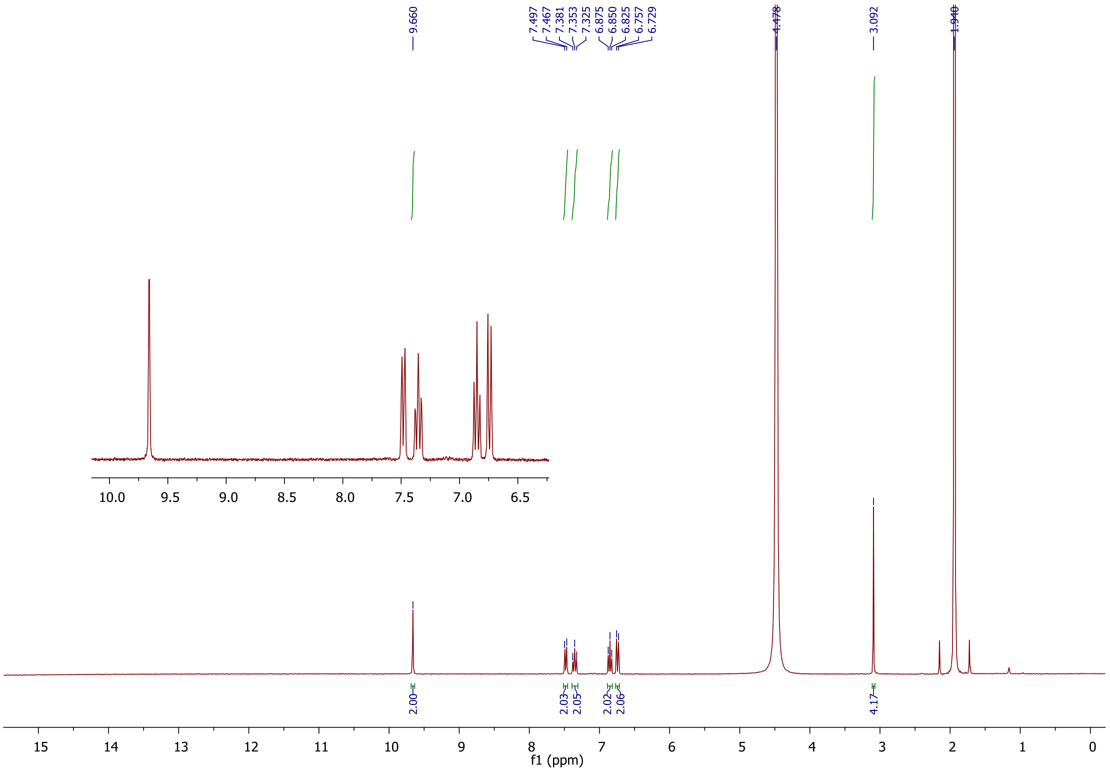

Figure S1. ^{1}H NMR spectrum of complex **3a** (300 MHz, CD_3CN).

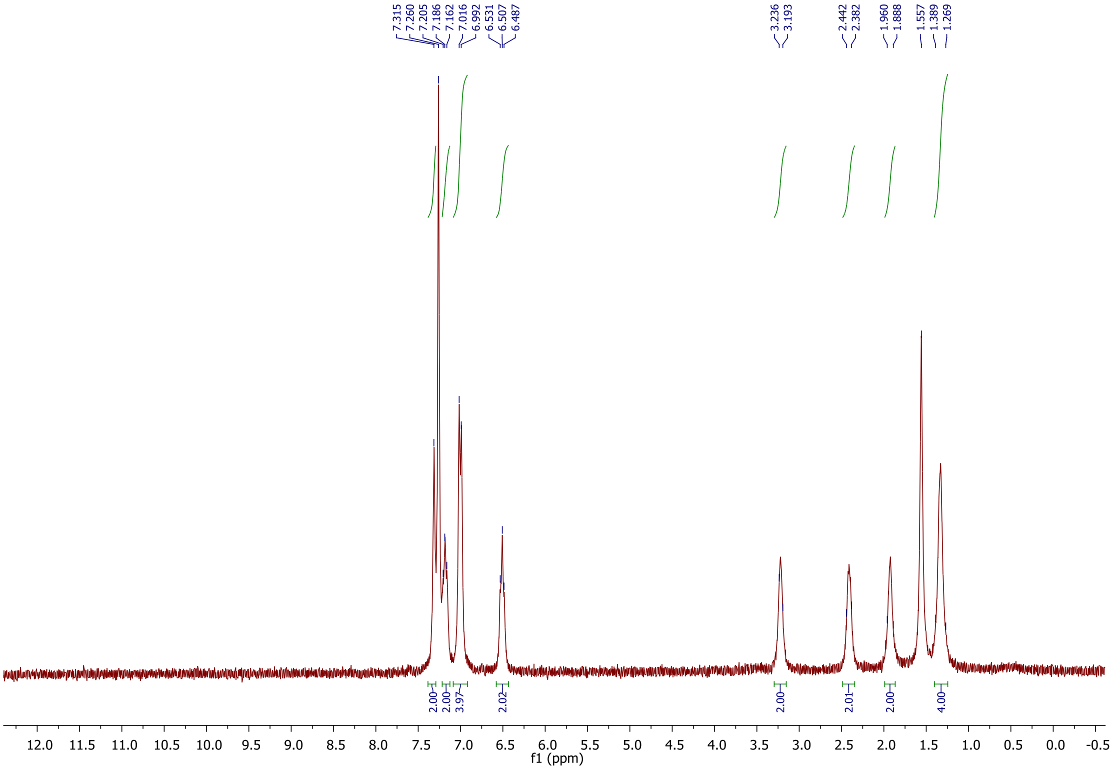

Figure S2. ^{1}H NMR spectrum of complex **3b** (300 MHz, $CDCl_3$).

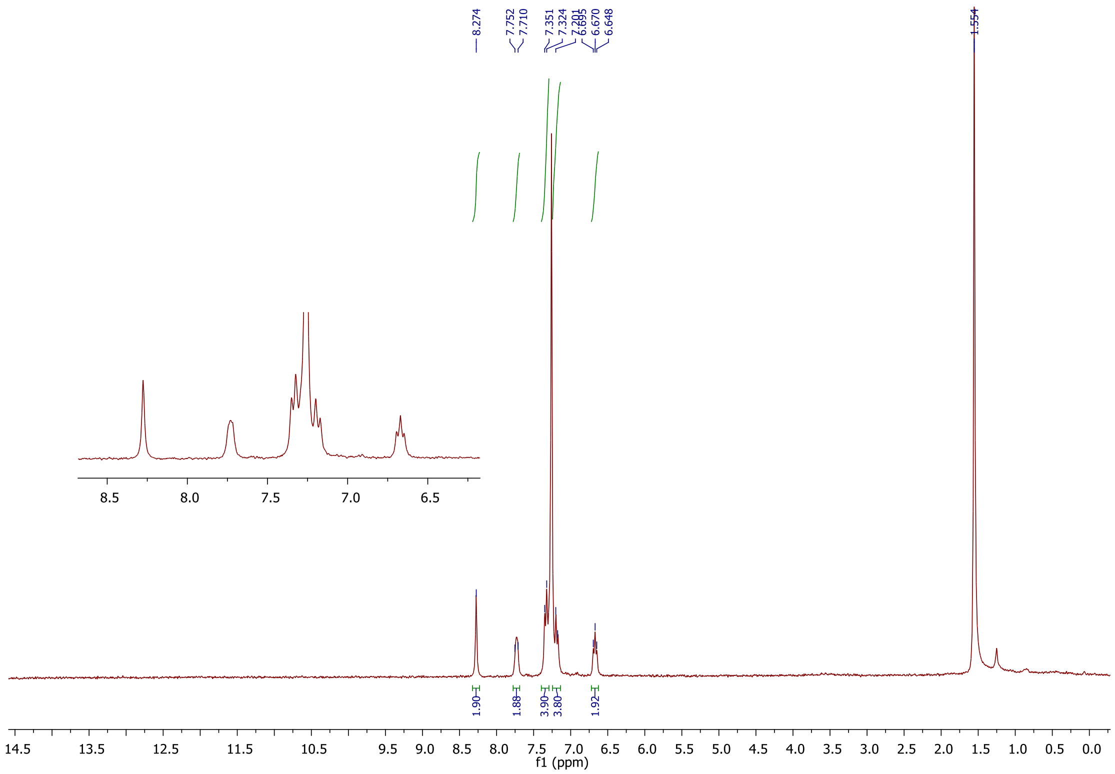

Figure S3. ^{1}H NMR spectrum of complex **3c** (300 MHz, $CDCl_3$).

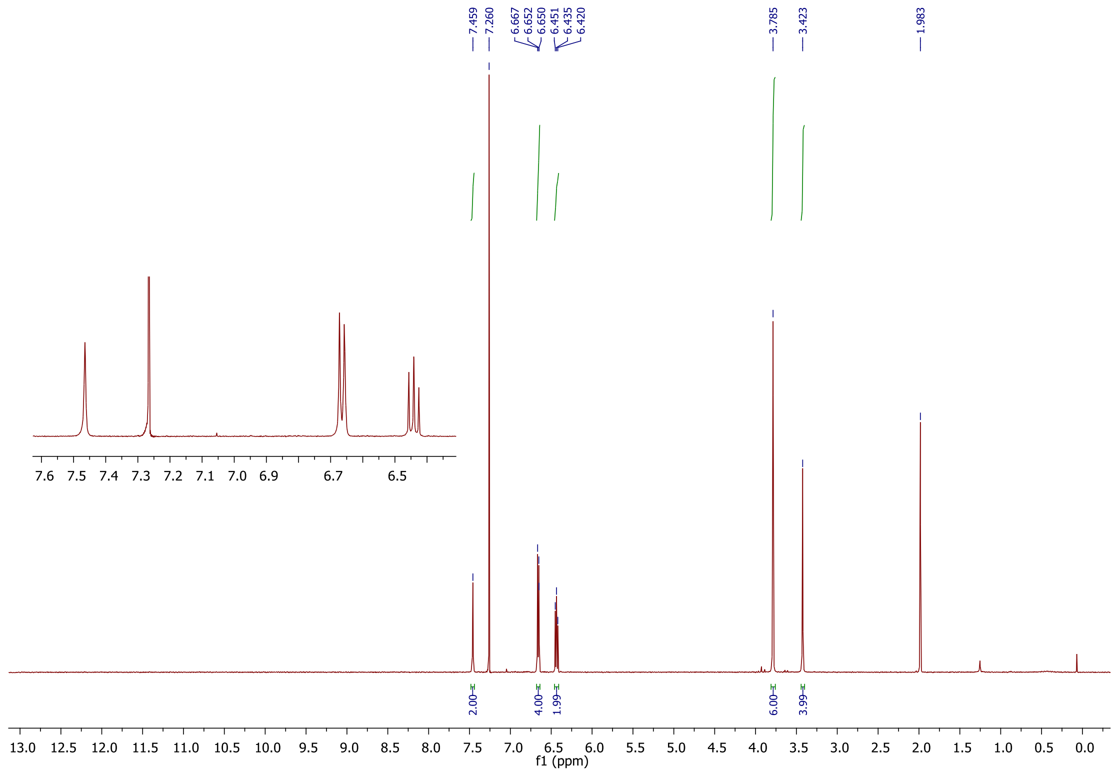

Figure S4. ^{1}H NMR spectrum of complex **3d** (500 MHz, $CDCl_3$).

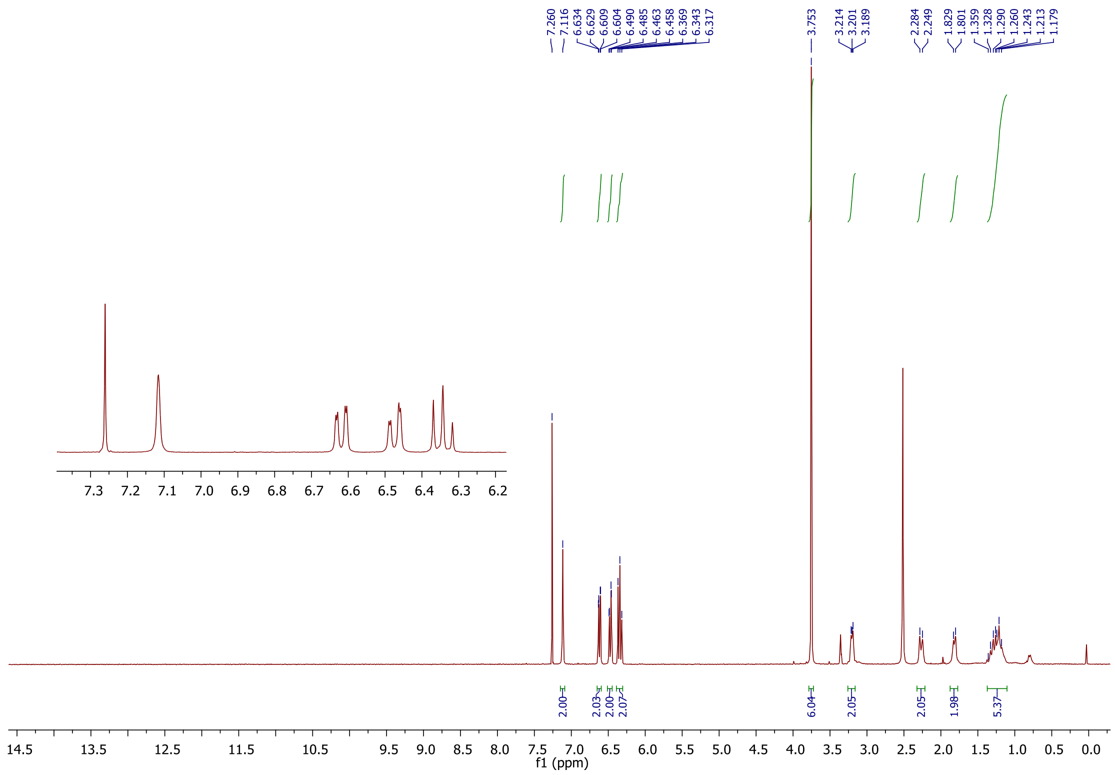

Figure S5. ^{1}H NMR spectrum of complex **3e** (300 MHz, $CDCl_3$).

12. ^{1}H NMR spectra of Zn^{II}-salen complexes, 4a-e

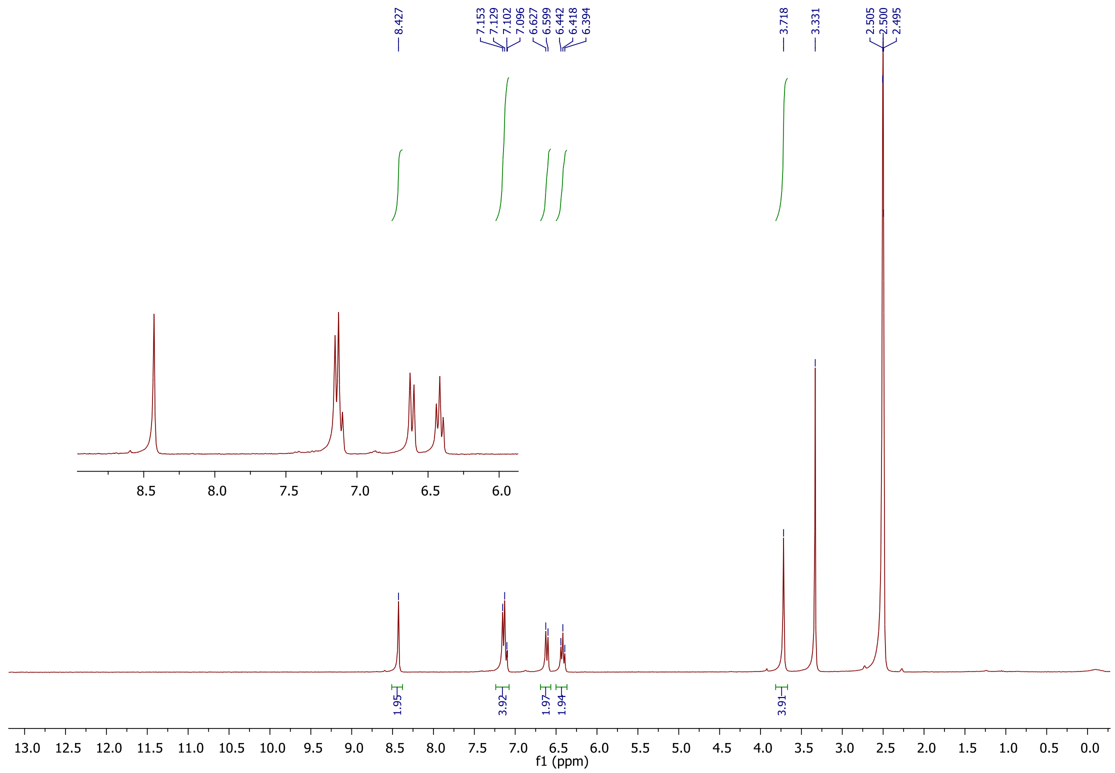

Figure S6. ^{1}H NMR spectrum of complex **4a** (500 MHz, d_6-DMSO).

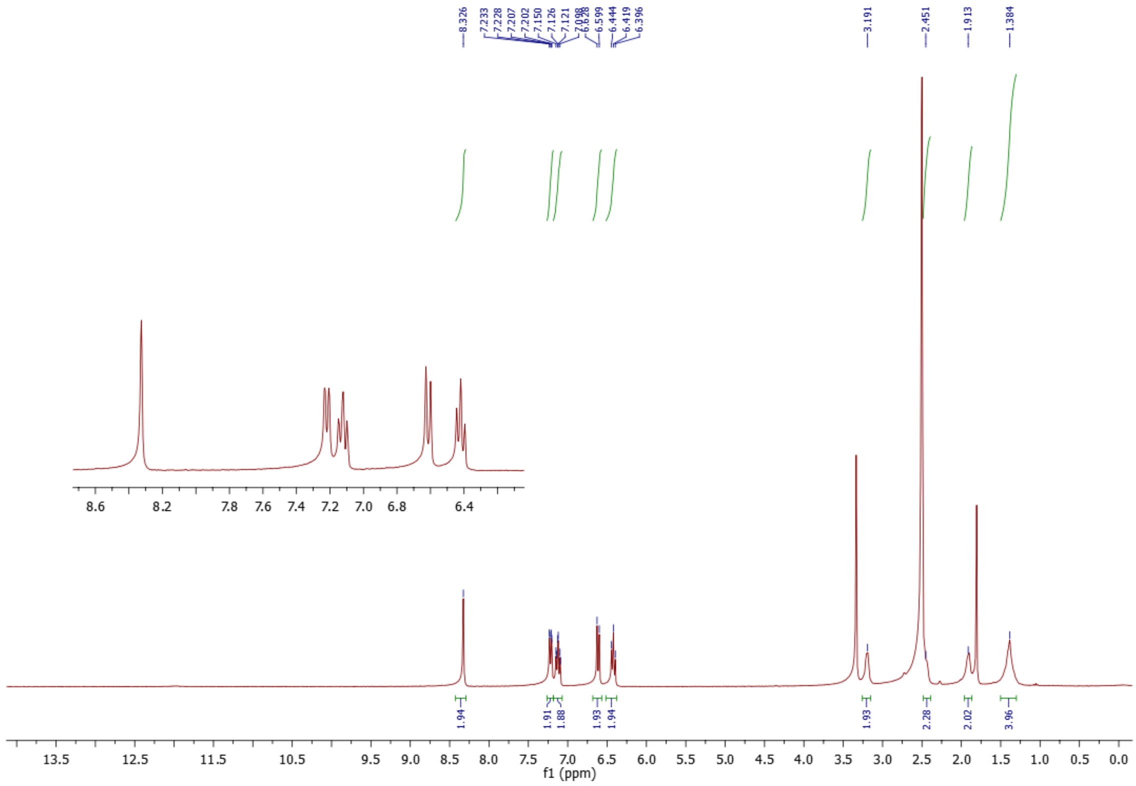

Figure S7. ^{1}H NMR spectrum of complex **4b** (500 MHz, d_6-DMSO).

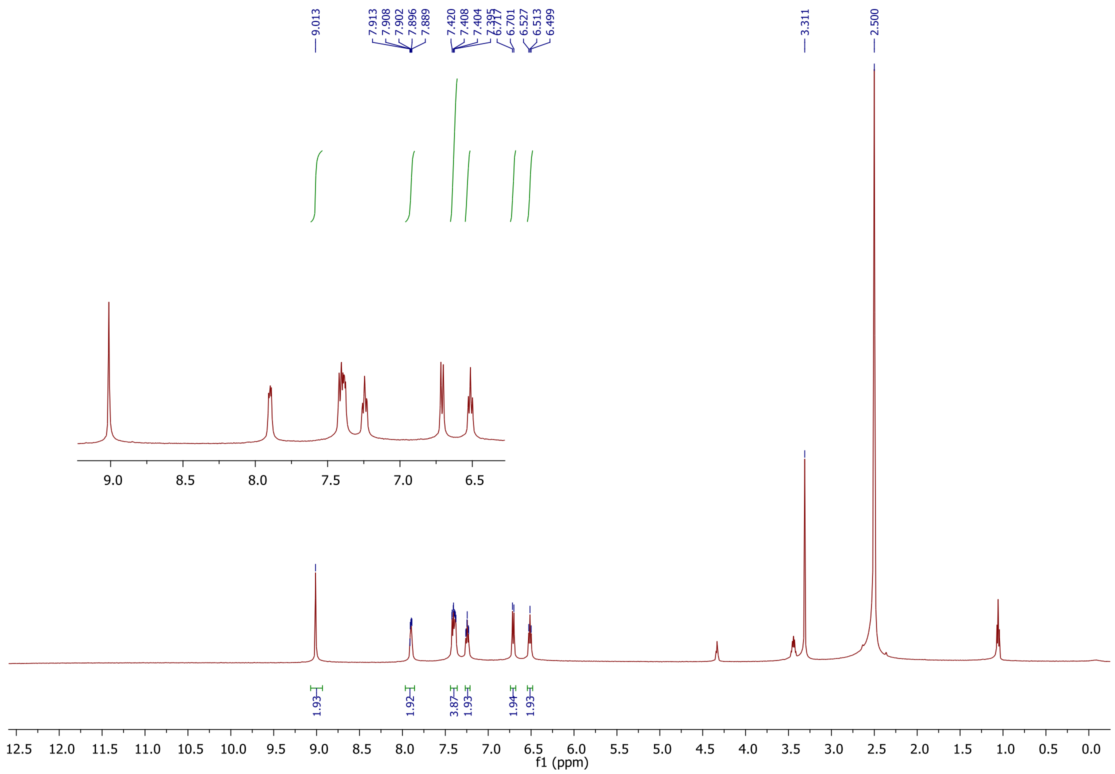

Figure S8. ^{1}H NMR spectrum of complex **4c** (500 MHz, d_6-DMSO).

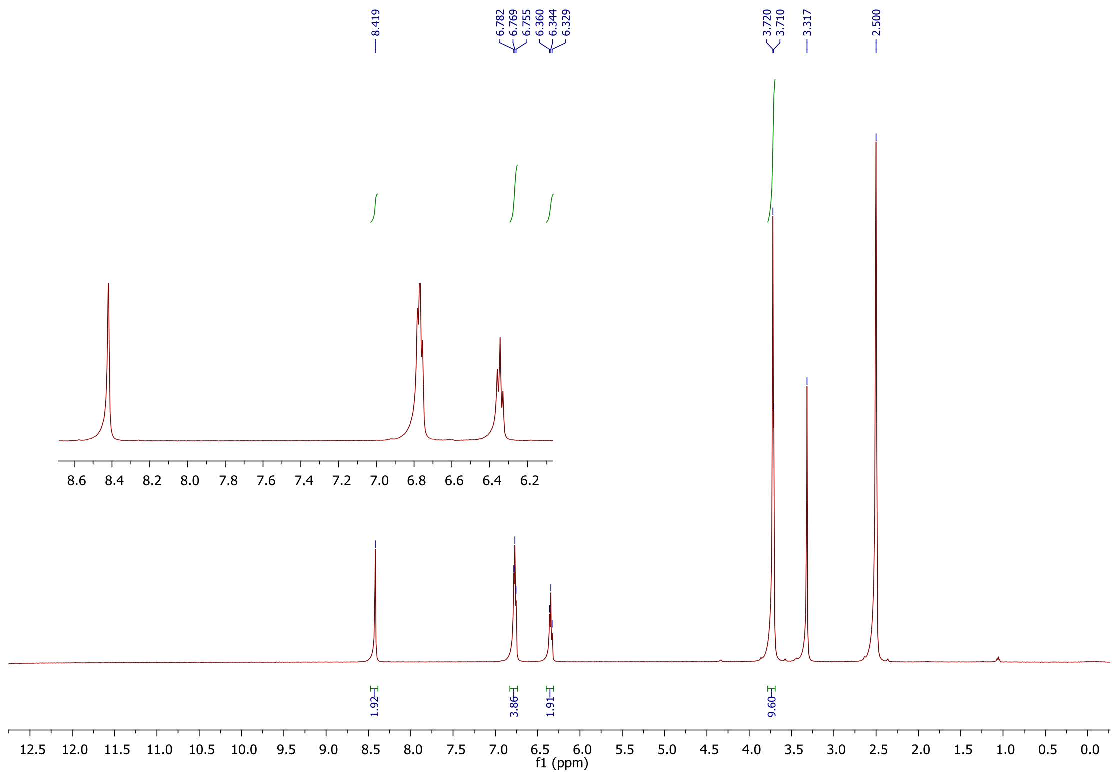

Figure S9. ^{1}H NMR spectrum of complex **4d** (500 MHz, d_6-DMSO).

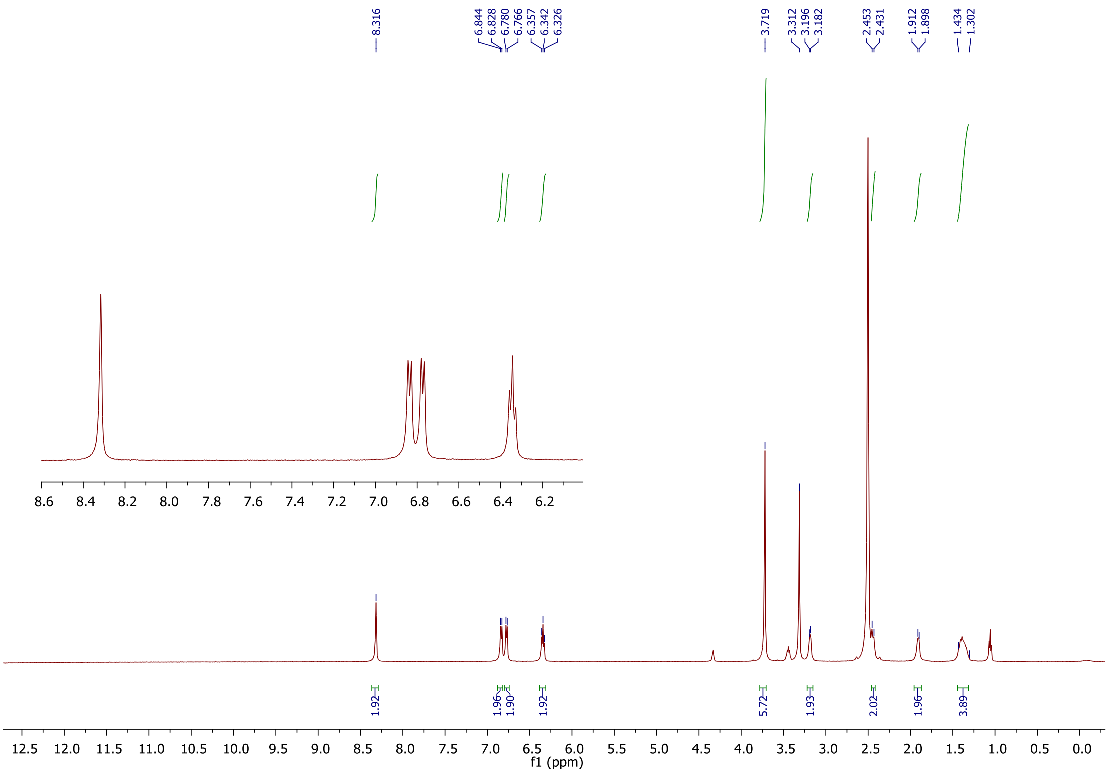

Figure S10. ^{1}H NMR spectrum of complex **4e** (500 MHz, d_6-DMSO).

13. Cyclic voltammetry measurements

Electrochemical measurements were conducted using an Autolab PGSTAT20 voltammetric analyser under an argon atmosphere, solvated in pre-dried/degassed CH_3CN containing 0.10 M [$^nBu_4N$]BF_4 as supporting electrolyte at room temperature. Voltammetric experiments utilised a Pt disk working electrode, a Pt rod auxiliary electrode and an Ag/AgCl reference electrode. All potentials quoted are referenced to an internal ferrocene/ferrocenium standard and were obtained at a scan rate (*v*) of 400 mVs^{-1}. The ferrocene/ferrocenium couple under these conditions was observed at $+0.40 \leq E_{1/2} \leq +0.55$ V vs Ag/AgCl.

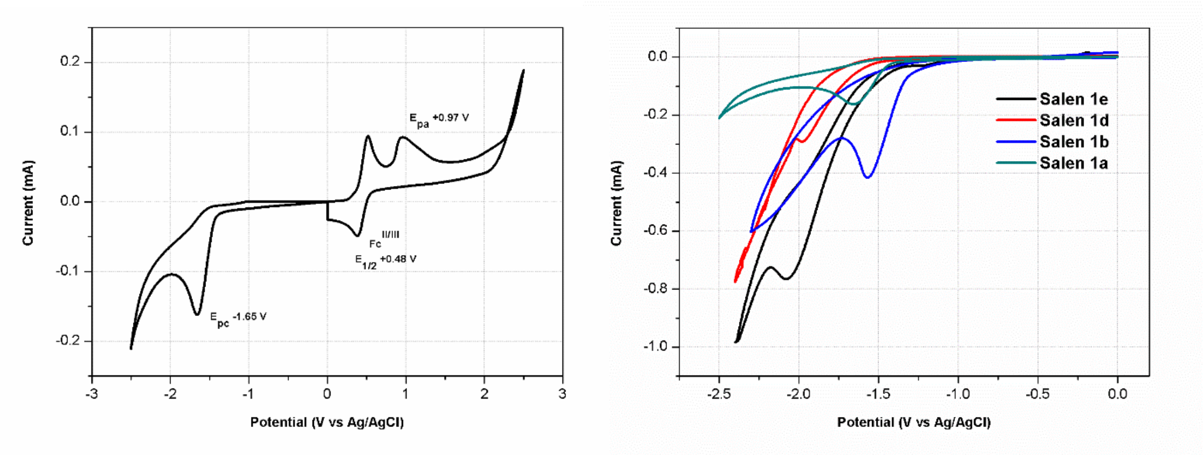

Figure S11. Full cyclic voltammogram of salen ligand **1c** (left) and cathodic sweep of salen ligands **1a**, **b**, **d**, **e** (right), illustrating regions of reduction potential in acetonitrile. [Salen ligand] = [Fc] = 1.0 mM; media = (CH_3CN/[$^nBu_4N$]BF_4 0.10 M); *v* = 400 mVs^{-1}; T = 298 K; E_{pa} ascribed to phenolate oxidation band.

Entry	Salen ligand peecursor	E_{red} *vs* Fc/Fc^+ (V)
1	1a	-2.12
2	1b	-2.04
3	1c	-2.13
4	1d	-2.45
5	1e	-2.55

Table S1. Reduction potentials (*versus* Fc/Fc^+) of salen ligand precursors **1a-e**.

14. Atomic absorption spectroscopy

Atomic absorption spectroscopy (AAS) was used to determine the presence (or absence) of elements (Mn and Ni) within bulk samples of **7a-c**, which were dissolved in 30 % nitric/10 % hydrochloric acid solution.

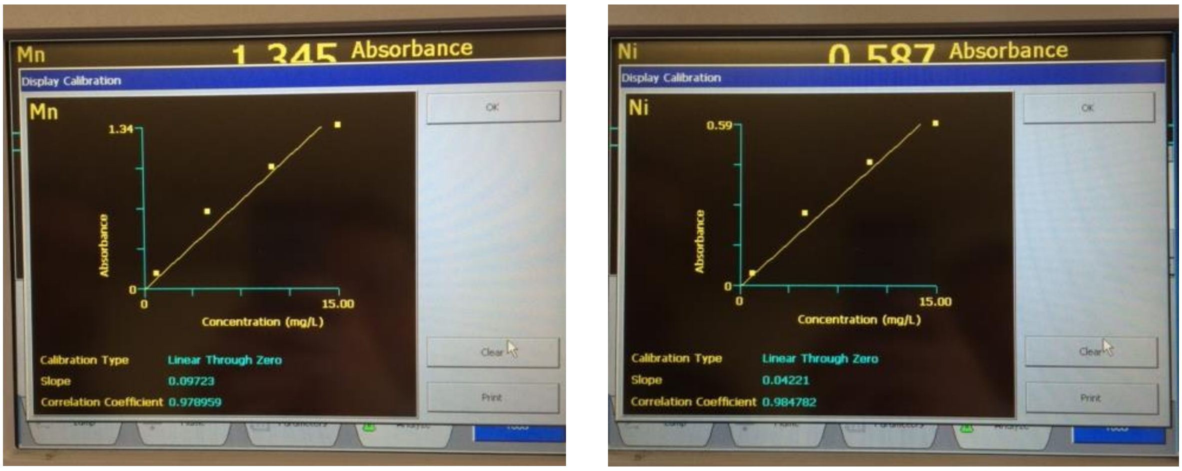

Figure S12. Atomic absorbance calibration profiles (1, 5, 10, 15 ppm stock solutions, diluted from 1000 ppm standard solutions using ultra-pure water) for Mn (left, correlation coefficient 0.98) and Ni (right, correlation coefficient 0.98).

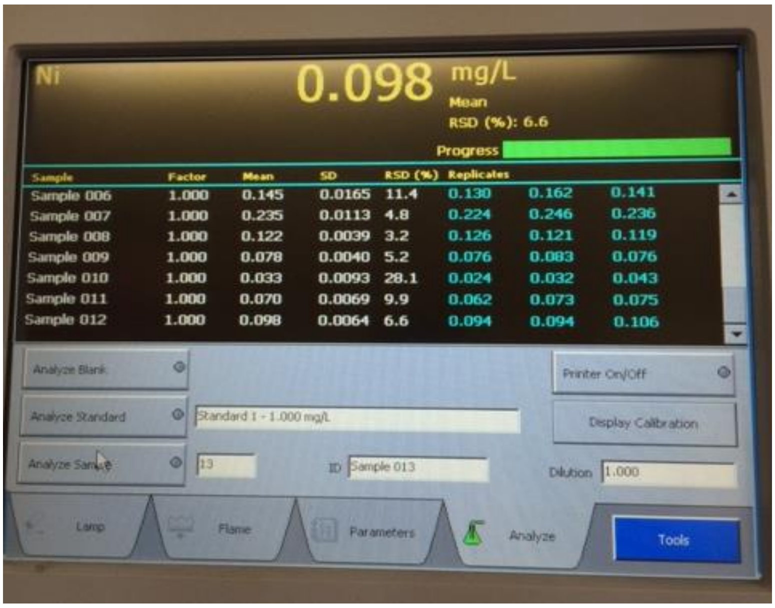

Figure S13. Atomic absorbance of Ni (Sample 009 = **7a**, 010 = **7b**, 011 = **7c**, 012 = **7a** at 10-fold concentration).

AAS analysis indicates trace quantities of Ni within bulk samples **7a-c** (Figure S13, **7a** = 0.076, **7b** = 0.043, **7c** = 0.075 ppm), which are consistent with quantities observed within blank solvent. A 10-fold increase in concentration of compound **7a** does not lead to a 10-fold increase in Ni absorbance (Figure S13, **7a** = 0.106 ppm), indicating that these readings lie within error associated with this instrument (*i.e.* no Ni above 1 ppm in any sample was accurately detected).

15. X-ray fluorescence analysis

All samples were run as solids on a Horiba XGT7000 X-ray analytical microscope instrument using a partial vacuum (X-ray voltage = 30 kV; preset time = 60 s; current set to auto; process time = 6; XGT beam size = 1.2 mm with no filter (F4) or Nb filter (F3). Solid samples were mounted on a glass slide and sealed using sellotape.

The first reference sample was benzylmanganese(I) pentacarbonyl (Figure S14) – data was collected at three separate positions to ensure that the sample was homogeneous.

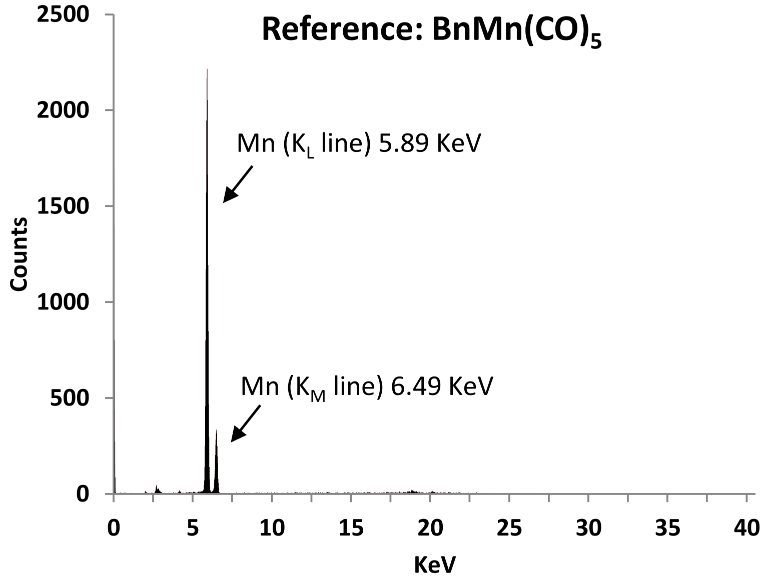

Figure S14. XRF analysis of benzylmanganese(I) pentacarbonyl (F4).

The second reference sample was $Ni(NO_3)_2 \cdot 6H_2O$ (Figure S15) – as the material was crystalline, one sample point was recorded with no filter (F4).

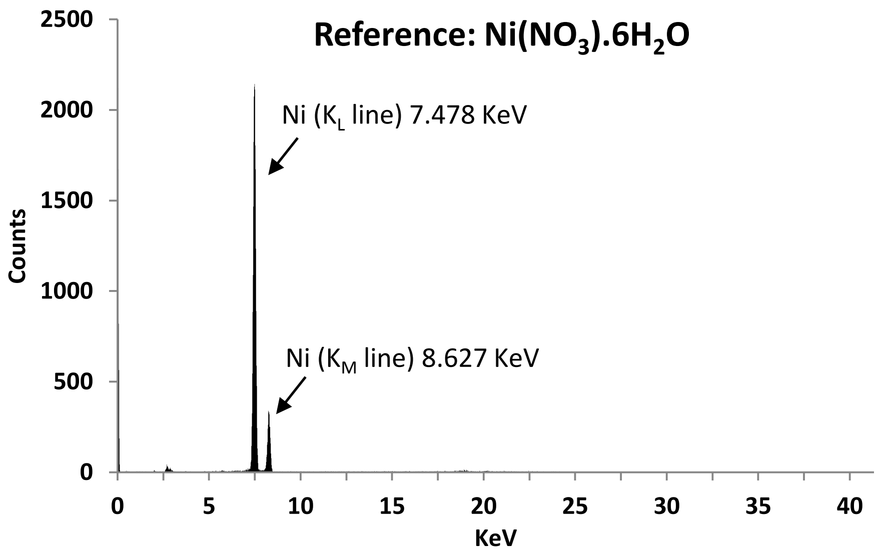

Figure S15. XRF analysis of $Ni(NO_3)_2{\cdot}6H_2O$ (F4).

The presence of Ni is indicated by characteristic K lines at 7.478 and 8.627 KeV (not present in the $BnMn(CO)_5$ reference sample).

Sample **7a** was analysed under the same conditions as for the reference samples (Figure S16). In total, six independent measurements were made to examine for sample homogeneity (with F3 applied). In all cases, Mn was the strongest element present and no Ni was detected (to the limits of detection ~ 10 ppm). For one sample point, trace Ca was detected (K lines at 4.013/3.690 KeV), when no filter (F4) was used.

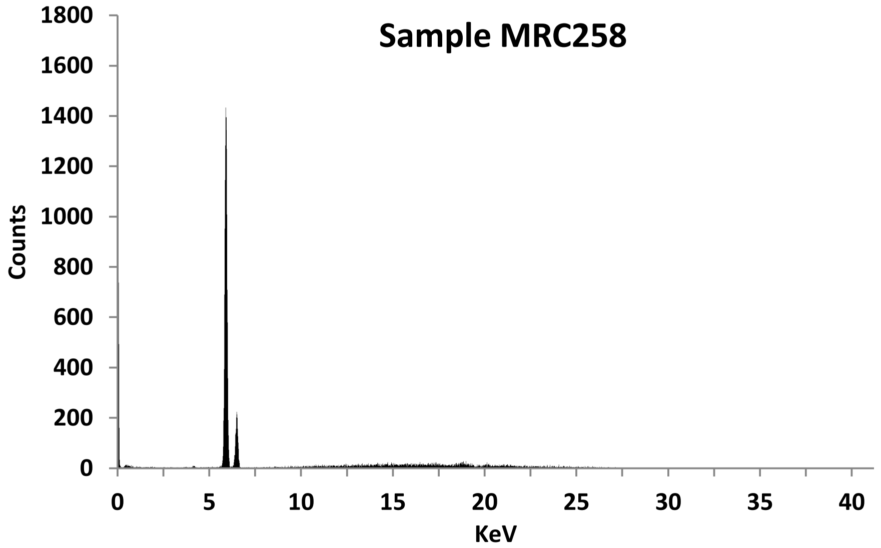

Figure S16. XRF analysis of sample **7a** (F3).

Sample **7b** was analysed under the same conditions as for the reference samples. In total, six independent measurements were made to examine the sample homogeneity. It was found that Ca and Cl (K lines at 2.816/2.621 KeV) were present in trace amounts when no filter (F4) was used (Figure S17). The Ca and Cl signals were lost using the F3 filter, with only Mn being visible (Figure S18). Ni was not found within this sample, to the limits of detection.

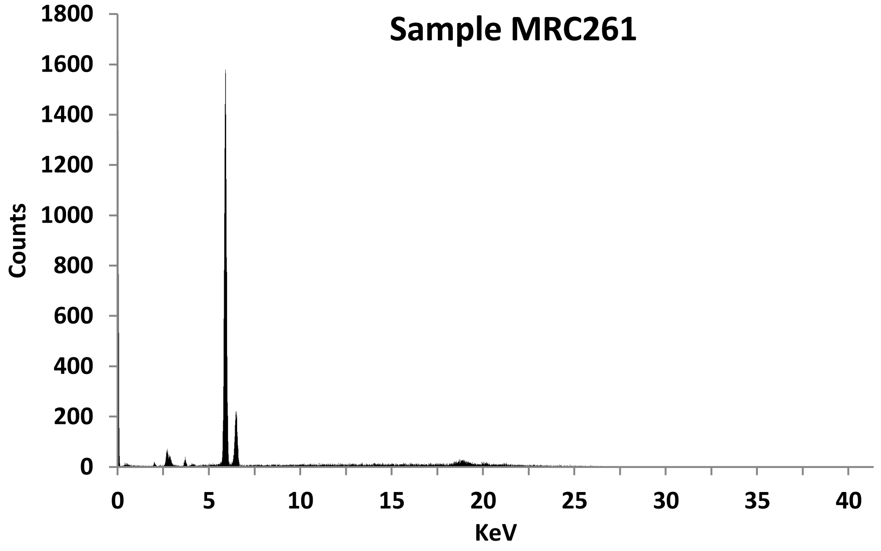

Figure S17. XRF analysis of sample **7b** (F4).

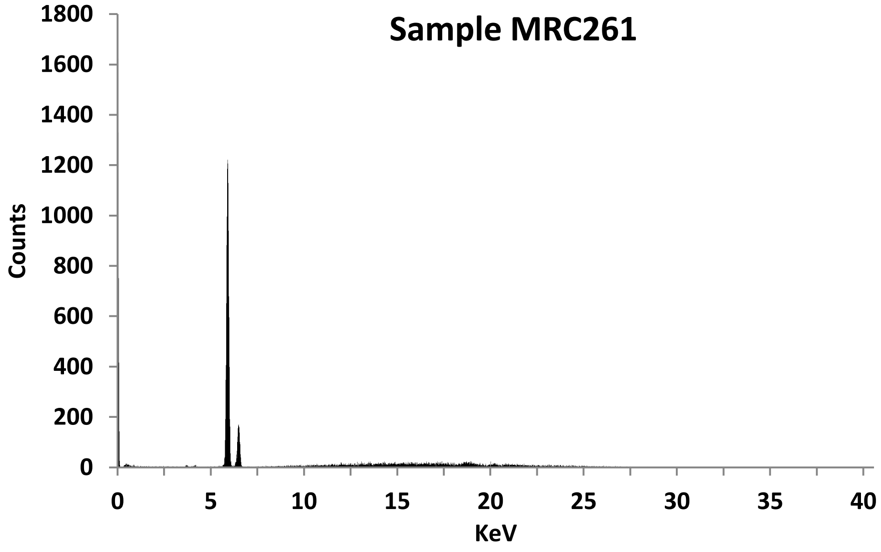

Figure S18. XRF analysis of sample **7b** (F3).

The analysis would indicate that trace CaCl is present in the sample. For quantification, it would be necessary to calibrate the analysis sample using authentic CaCl samples.

Sample **7c** was analysed under the same conditions as for the reference samples. In total, five independent measurements were made to examine the sample homogeneity. For two of the five samples, a trace signal that may be indicative of Ca, was observed when no filter (F4) was used in the measurement (Figure S19), which was lost when the Nb filter (F3) was used (Figure S20). In all cases, the Mn signal was dominant. Ni was not found within this sample, to the limits of detection.

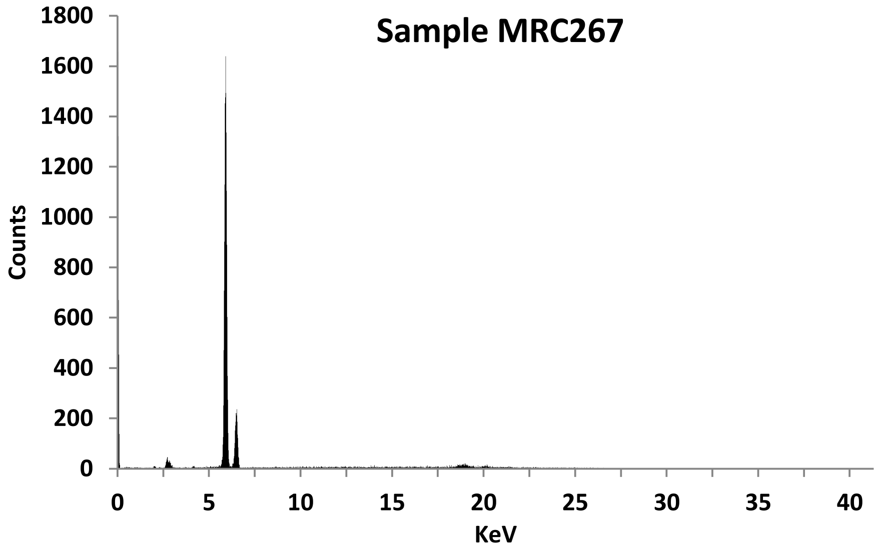

Figure S19. XRF analysis of sample **7c** (F4).

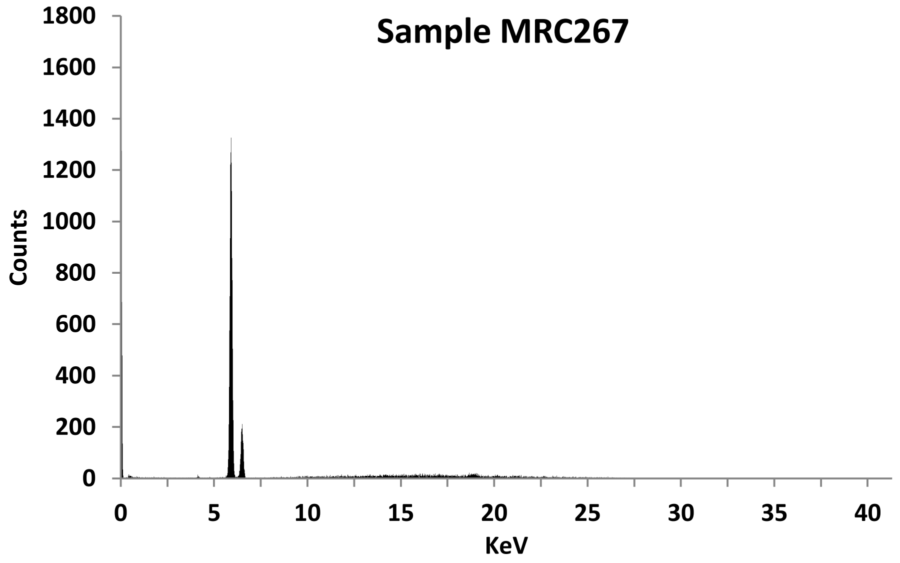

Figure S20. XRF analysis of sample **7c** (F3).

16. Magnetic susceptibility measurements

The solution magnetic susceptibility measurements were made by NMR methods on a Bruker AV500 spectrometer at 298 K using a modified Evans method.[6] A flame-sealed coaxial insert containing a deuterated reference solvent (C_6D_6/C_6H_6, 50:50) was placed in a Young's NMR tube under an inert atmosphere. An anhydrous (anoxic) C_6D_6 (100 %) solution of the paramagnetic complex was added to the tube and $^1H\{^{13}C\}$ NMR spectra recorded. The volume magnetic susceptibility (χ) of the paramagnetic compound was calculated from the following equation:

$$\chi = \chi_0 + \frac{3000\Delta\nu}{4\pi\nu_0 cM}$$

where χ_0 is the volume magnetic susceptibility of pure solvent; $\Delta\nu$ (Hz) is the separation of the reference solvent peaks from the inner and outer tubes; ν_0 is the operating frequency of the spectrometer; c is the concentration of the complex in $molL^{-1}$ and M is the molecular weight of the complex. The molar susceptibility (χ_M) and magnetic moment (μ_{eff}) were calculated from χ at 298 K, taking account of the diamagnetic correction from the sum of Pascal constants.[7]

17. Electrochemical reactor configuration

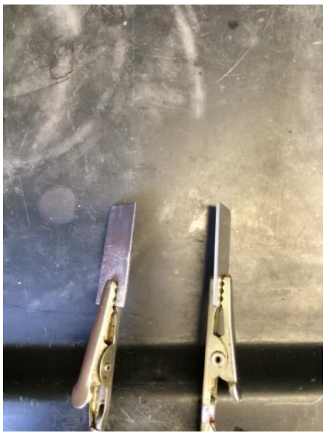

(a)

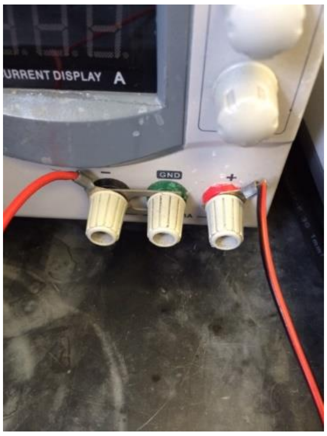

(b)

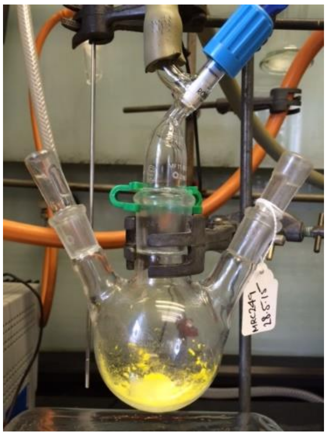

(c)

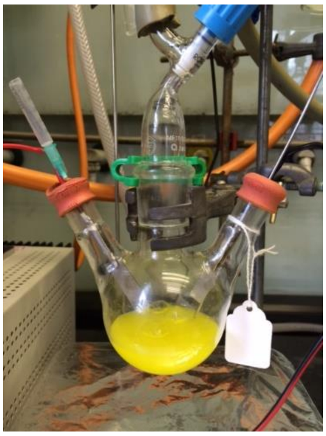

(d)

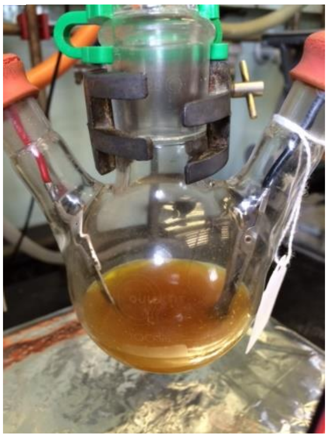

(e)

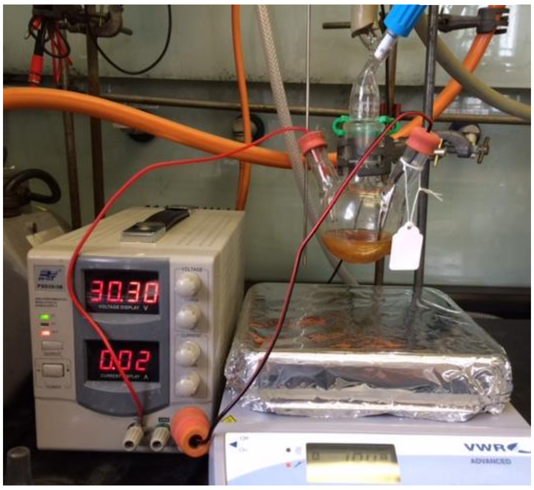

(f)

Inert procedure:

A pair of oven-dried metal electrodes are each independently attached to standard electrical wire (**a**). Each electrode is independently connected to a positive and negative terminal of a power supply (**b**). A flame-dried three-necked flask is charged with salen precursor, and further dried *in vacuo* for 30 minutes (**c**). Anhydrous (and anoxic) acetonitrile is added to the flask, and further degassed *via* bubbling a flow of argon through the solution for 30 minutes (**d**). Whilst under an inert atmosphere, the two electrodes are introduced to the solution (**e**) and a potential applied to the cell (**f**) to maintain a constant current (20 – 50 mA).

Hydrous procedure:

A pair of metal electrodes are prepared as above. A three-necked flask is charged with salen precursor, dissolved with HPLC-grade acetonitrile and both electrodes are introduced to the solution. Whilst under an atmosphere of air, a potential is applied to the cell (see above).

18. Crystallographic details

X-Ray diffraction data were collected on an Agilent SuperNova diffractometer fitted with an Atlas CCD detector with Mo K*α* radiation (λ = 0.7107 Å) or Cu K*α* radiation (λ = 1.5418 Å). Crystals were mounted under oil on nylon fibres. Data sets were corrected for absorption using a multiscan method, and the structures were solved by direct methods using SHELXS-97/SHELXT and refined by full-matrix least squares on F^2 using ShelXL-97, interfaced through the program Olex2.[8] Molecular graphics for all structures were generated using POV-RAY in the X-Seed program.

Crystallographic details for 2g:

Identification code	SEH30_Cu
Empirical formula	$C_{38}H_{62}CuN_2O_2$
Formula weight	642.44
Temperature/K	120.3(7)
Crystal system	orthorhombic
Space group	Pbca
a/Å	17.7387(4)
b/Å	11.6590(2)
c/Å	34.8626(6)
α/°	90.00
β/°	90.00
γ/°	90.00
Volume/Å^3	7210.2(2)
Z	8
ρ_{calc}g/cm^3	1.184
μ/mm^{-1}	1.096
F(000)	2792.0
Crystal size/mm^3	0.33 × 0.04 × 0.03
Radiation	CuKα (λ = 1.54184)
2Θ range for data collection/°	7.1 to 134.06
Index ranges	-20 ≤ h ≤ 21, -13 ≤ k ≤ 13, -41 ≤ l ≤ 29
Reflections collected	18677
Independent reflections	6424 [R_{int} = 0.0321, R_{sigma} = 0.0325]
Data/restraints/parameters	6424/0/402
Goodness-of-fit on F^2	1.036
Final R indexes [I>=2σ (I)]	R_1 = 0.0548, wR_2 = 0.1465
Final R indexes [all data]	R_1 = 0.0649, wR_2 = 0.1563
Largest diff. peak/hole / e Å^{-3}	1.76/-0.66

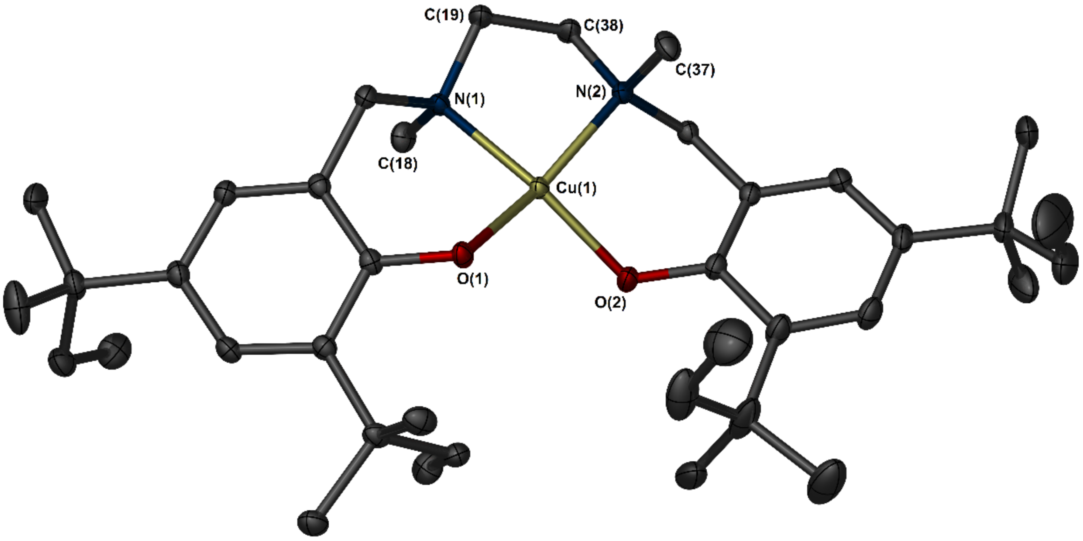

Figure S22. Molecular structure of complex **2g**.

Crystallographic details for 2h:

Identification code	SEH31_Cu
Empirical formula	$C_{34}H_{54}CuN_2O_2$
Formula weight	586.33
Temperature/K	121(1)
Crystal system	orthorhombic
Space group	Pbca
a/Å	17.3203(10)
b/Å	10.9924(11)
c/Å	34.278(2)
α/°	90.00
β/°	90.00
γ/°	90.00
Volume/Å^3	6526.2(9)
Z	8
ρ_{calc}g/cm^3	1.193
μ/mm^{-1}	1.166
F(000)	2536.0
Crystal size/mm^3	0.27 × 0.03 × 0.02
Radiation	CuKα (λ = 1.54184)
2Θ range for data collection/°	7.26 to 134.12
Index ranges	$-18 \leq h \leq 20$, $-13 \leq k \leq 12$, $-28 \leq l \leq 40$
Reflections collected	15600
Independent reflections	5824 [R_{int} = 0.1069, R_{sigma} = 0.1173]
Data/restraints/parameters	5824/0/366

Goodness-of-fit on F^2	0.974
Final R indexes [I>=2σ (I)]	R_1 = 0.0643, wR_2 = 0.1401
Final R indexes [all data]	R_1 = 0.1223, wR_2 = 0.1713
Largest diff. peak/hole / e $Å^{-3}$	0.79/-0.75

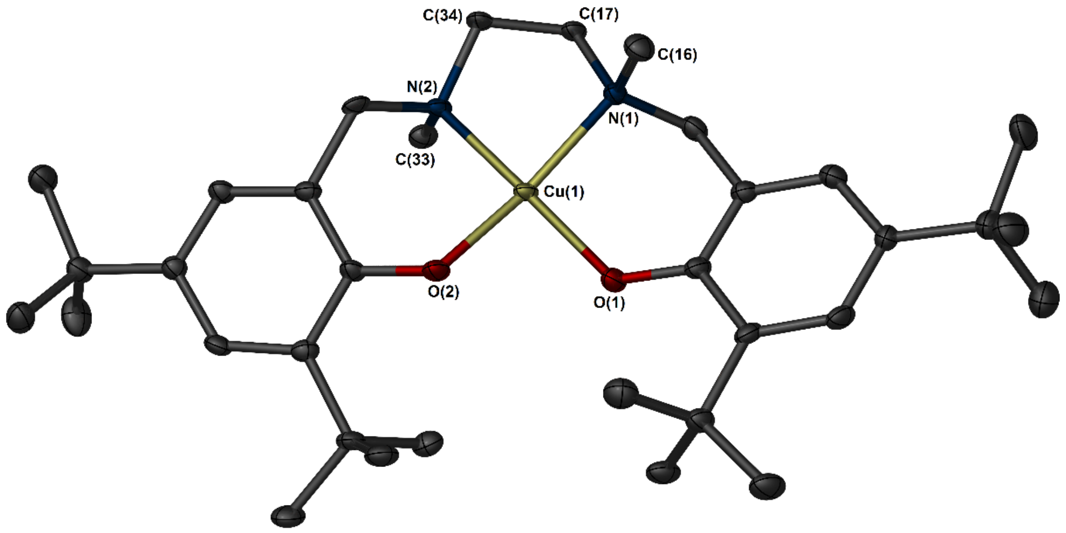

Figure S23. Molecular structure of complex **2h**.

Crystallographic details for 3b:

Identification code	MRC236_Cu
Empirical formula	$C_{20}H_{20}N_2NiO_2$
Formula weight	379.09
Temperature/K	120.01(13)
Crystal system	orthorhombic
Space group	Pnna
a/Å	6.8080(4)
b/Å	13.6311(7)
c/Å	17.8457(11)
α/°	90.00
β/°	90.00
γ/°	90.00
Volume/$Å^3$	1656.10(16)
Z	4
ρ_{calc}g/cm^3	1.520
μ/mm^{-1}	1.818
F(000)	792.0
Crystal size/mm^3	0.3738 × 0.117 × 0.03
Radiation	CuKα (λ = 1.54184)
2Θ range for data collection/°	8.16 to 146.82

Index ranges	-8 ≤ h ≤ 4, -16 ≤ k ≤ 15, -21 ≤ l ≤ 14
Reflections collected	3881
Independent reflections	1630 [R_{int} = 0.0406, R_{sigma} = 0.0431]
Data/restraints/parameters	1630/0/114
Goodness-of-fit on F^2	1.038
Final R indexes [I>=2σ (I)]	R_1 = 0.0442, wR_2 = 0.1150
Final R indexes [all data]	R_1 = 0.0586, wR_2 = 0.1258
Largest diff. peak/hole / e $Å^{-3}$	0.48/-0.45

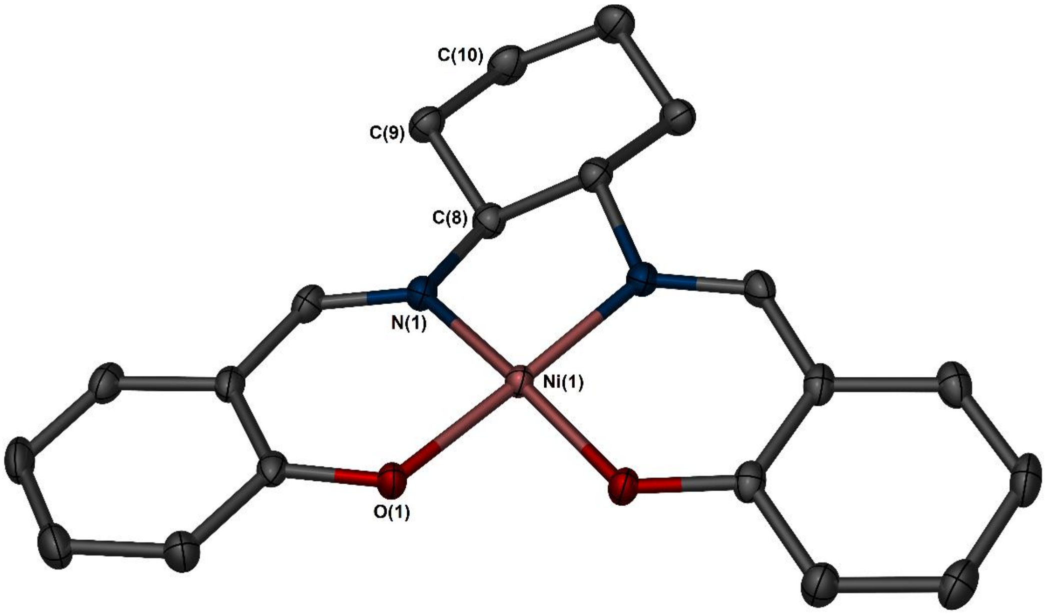

Figure S24. Molecular structure of complex **3b**.

Crystallographic details for 3e:

Identification code	seh38_cu_sqd
Empirical formula	$C_{23.75}H_{23}N_2NiO_{4.5}$
Formula weight	467.15
Temperature/K	120.2(5)
Crystal system	orthorhombic
Space group	Cmcm
a/Å	18.9750(12)
b/Å	16.8021(10)
c/Å	6.8430(5)
α/°	90.00
β/°	90.00
γ/°	90.00
Volume/$Å^3$	2181.7(2)
Z	4
ρ_{calc}g/cm^3	1.422

μ/mm^{-1}	0.925
F(000)	974.0
Crystal size/mm^3	0.1390 × 0.0333 × 0.0220
Radiation	MoKα (λ = 0.71073)
2Θ range for data collection/°	3.24 to 52.56
Index ranges	$-21 \leq h \leq 22$, $-20 \leq k \leq 19$, $-8 \leq l \leq 7$
Reflections collected	4067
Independent reflections	1225 [R_{int} = 0.0565, R_{sigma} = 0.0420]
Data/restraints/parameters	1225/0/97
Goodness-of-fit on F^2	1.124
Final R indexes [I>=2σ (I)]	R_1 = 0.0621, wR_2 = 0.1723
Final R indexes [all data]	R_1 = 0.0708, wR_2 = 0.1810
Largest diff. peak/hole / e Å^{-3}	0.78/-0.37

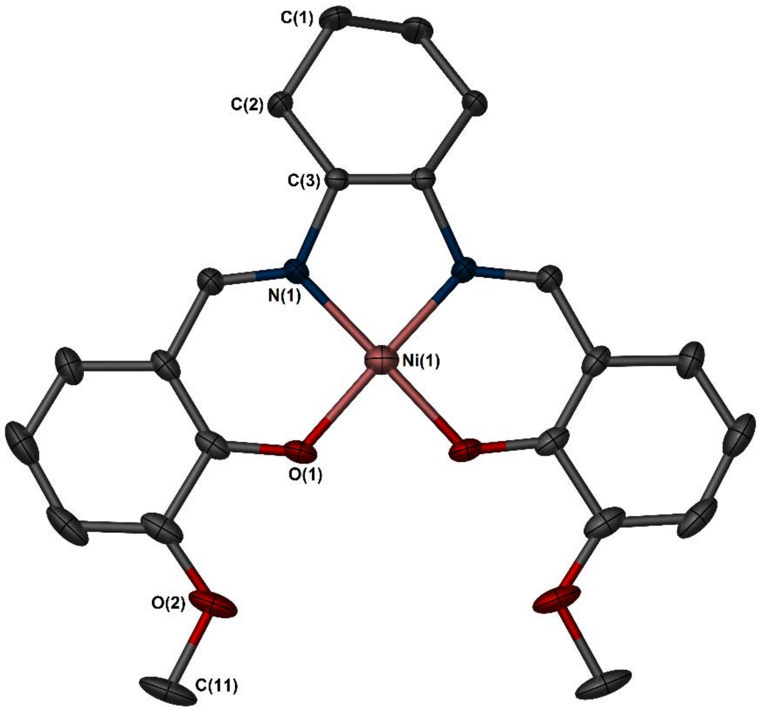

Figure S25. Molecular structure of complex **3e**.

Crystallographic details for 4e:

Identification code	MRC240
Empirical formula	$C_{24}H_{30}N_2O_5SZn$
Formula weight	523.93
Temperature/K	120.01(10)
Crystal system	triclinic
Space group	P-1
a/Å	9.6395(10)
b/Å	11.5180(6)

c/Å	11.8273(10)
α/°	104.074(6)
β/°	113.640(9)
γ/°	92.856(7)
Volume/Å^3	1150.69(17)
Z	2
ρ_{calc}g/cm^3	1.512
μ/mm^{-1}	1.197
F(000)	548.0
Crystal size/mm^3	0.3594 × 0.2312 × 0.1012
Radiation	MoKα (λ = 0.71073)
2Θ range for data collection/°	6.12 to 62.52
Index ranges	-12 ≤ h ≤ 12, -15 ≤ k ≤ 16, -17 ≤ l ≤ 16
Reflections collected	13919
Independent reflections	6502 [R_{int} = 0.0538, R_{sigma} = 0.0853]
Data/restraints/parameters	6502/0/309
Goodness-of-fit on F^2	1.062
Final R indexes [I>=2σ (I)]	R_1 = 0.0596, wR_2 = 0.1279
Final R indexes [all data]	R_1 = 0.0841, wR_2 = 0.1456
Largest diff. peak/hole / e Å^{-3}	1.57/-0.49

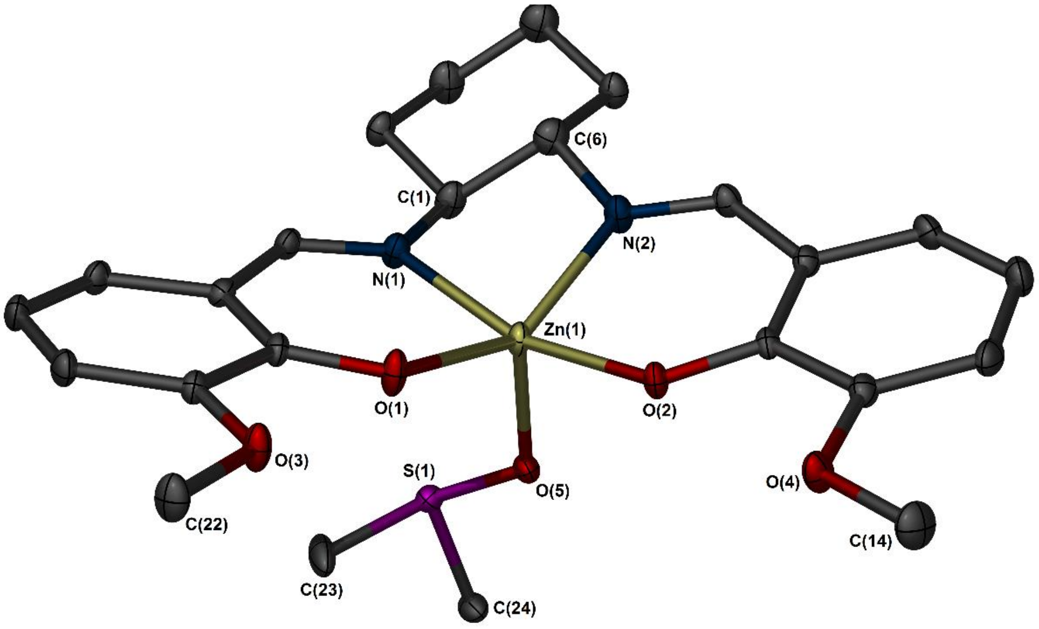

Figure S26. Molecular structure of complex **4e**.

Crystallographic details for 5a:

Identification code	MRC35(2_Cu)
Empirical formula	$C_{64}H_{56}Fe_4N_8O_{10}$
Formula weight	1320.57

Temperature/K	120.1(4)
Crystal system	triclinic
Space group	P-1
a/Å	10.8736(5)
b/Å	11.0146(5)
c/Å	13.7145(7)
α/°	67.507(5)
β/°	72.885(4)
γ/°	76.765(4)
Volume/Å^3	1437.75(11)
Z	1
ρ_{calc}g/cm^3	1.525
μ/mm^{-1}	8.500
F(000)	680.0
Crystal size/mm^3	0.3853 × 0.1701 × 0.0575
Radiation	CuKα (λ = 1.54184)
2Θ range for data collection/°	8.58 to 147.66
Index ranges	$-13 \leq h \leq 13$, $-12 \leq k \leq 10$, $-17 \leq l \leq 13$
Reflections collected	10707
Independent reflections	5434 [R_{int} = 0.0418, R_{sigma} = 0.0485]
Data/restraints/parameters	5434/0/388
Goodness-of-fit on F^2	1.031
Final R indexes [I>=2σ (I)]	R_1 = 0.0530, wR_2 = 0.1458
Final R indexes [all data]	R_1 = 0.0568, wR_2 = 0.1507
Largest diff. peak/hole / e Å^{-3}	1.57/-0.53

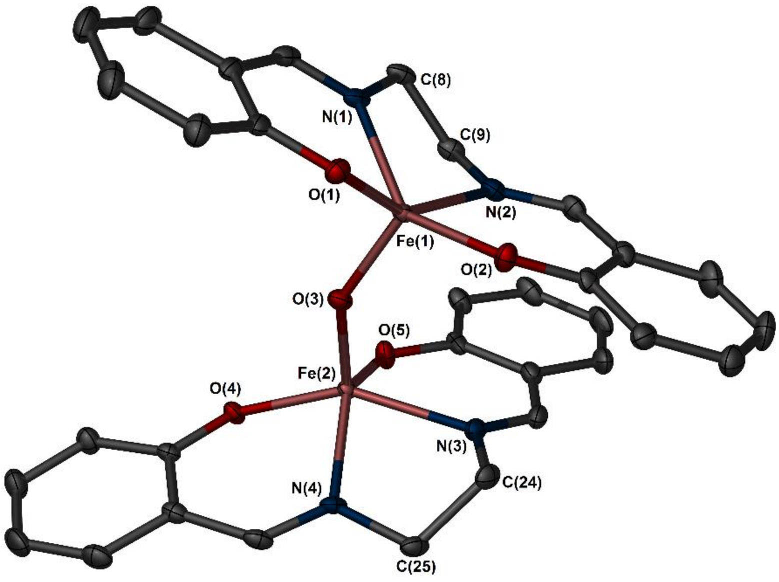

Figure S27. Molecular structure of complex **5a**.

Crystallographic details for 6e:

Identification code	MRC250_Cu(3)_twin1_hklf4
Empirical formula	$C_{22}H_{28}FeN_2O_5$
Formula weight	456.31
Temperature/K	120.01(14)
Crystal system	monoclinic
Space group	$P2_1/n$
a/Å	13.356(4)
b/Å	7.725(2)
c/Å	19.505(5)
α/°	90
β/°	93.71(3)
γ/°	90
Volume/Å^3	2008.3(9)
Z	4
ρ_{calc}g/cm^3	1.509
μ/mm^{-1}	0.789
F(000)	960.0
Crystal size/mm^3	0.211 × 0.0601 × 0.0395
Radiation	MoKα (λ = 0.71073)
2Θ range for data collection/°	3.59 to 44.198
Index ranges	$-14 \leq h \leq 14$, $-8 \leq k \leq 8$, $-19 \leq l \leq 20$
Reflections collected	3516
Independent reflections	3516 [R_{int} = ?, R_{sigma} = 0.1667]
Data/restraints/parameters	3516/330/313
Goodness-of-fit on F^2	0.978
Final R indexes [I>=2σ (I)]	R_1 = 0.0898, wR_2 = 0.2015
Final R indexes [all data]	R_1 = 0.1594, wR_2 = 0.2268
Largest diff. peak/hole / e Å^{-3}	1.00/-0.42

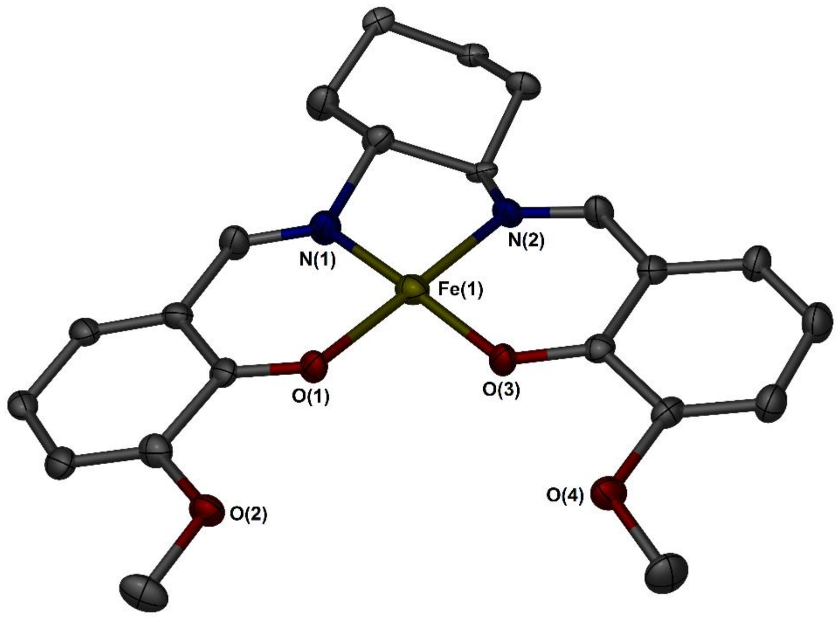

Figure S28. Molecular structure of complex **6e**.

Crystallographic details for 7a:

Identification code	exp_1330
Empirical formula	$C_{17}H_{16}Cl_2MnN_2O_3$
Formula weight	422.16
Temperature/K	120.00(10)
Crystal system	monoclinic
Space group	$P2_1/c$
a/Å	9.7531(3)
b/Å	8.3562(2)
c/Å	21.8483(6)
α/°	90.00
β/°	100.943(3)
γ/°	90.00
Volume/Å^3	1748.24(8)
Z	4
ρ_{calc}g/cm^3	1.604
μ/mm^{-1}	1.080
F(000)	860.0
Crystal size/mm^3	0.0829 × 0.0653 × 0.0414
Radiation	MoKα (λ = 0.71073)
2Θ range for data collection/°	4.26 to 52.56
Index ranges	$-12 \le h \le 11$, $-5 \le k \le 10$, $-21 \le l \le 26$
Reflections collected	6574
Independent reflections	3411 [R_{int} = 0.0420, R_{sigma} = 0.0468]

Data/restraints/parameters	3411/0/226
Goodness-of-fit on F^2	1.197
Final R indexes [I>=2σ (I)]	R_1 = 0.1234, wR_2 = 0.2728
Final R indexes [all data]	R_1 = 0.1298, wR_2 = 0.2845
Largest diff. peak/hole / e $Å^{-3}$	1.99/-0.81

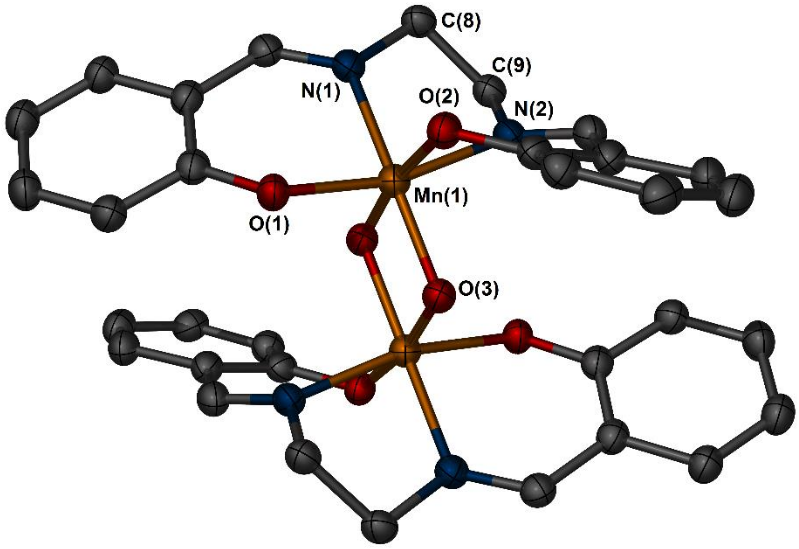

Figure S29. Molecular structure of complex **7a**.

Crystallographic details for 7a:

Identification code	MRC258_bis salen
Empirical formula	$C_{36}H_{40}Mn_2N_4O_8S_2$
Formula weight	830.72
Temperature/K	120.01(10)
Crystal system	monoclinic
Space group	$P2_1/n$
a/Å	6.2918(2)
b/Å	14.1567(5)
c/Å	20.0004(7)
α/°	90.00
β/°	92.700(3)
γ/°	90.00
Volume/$Å^3$	1779.47(11)
Z	2
$\rho_{calc}g/cm^3$	1.550
μ/mm^{-1}	0.886
F(000)	860.0
Crystal size/mm^3	0.281 × 0.242 × 0.0318
Radiation	MoKα (λ = 0.71073)
2Θ range for data collection/°	6.7 to 62.64

Index ranges	$-6 \leq h \leq 9$, $-19 \leq k \leq 13$, $-28 \leq l \leq 28$
Reflections collected	13057
Independent reflections	5065 [R_{int} = 0.0433, R_{sigma} = 0.0571]
Data/restraints/parameters	5065/0/244
Goodness-of-fit on F^2	1.032
Final R indexes [I>=2σ (I)]	R_1 = 0.0474, wR_2 = 0.0970
Final R indexes [all data]	R_1 = 0.0685, wR_2 = 0.1059
Largest diff. peak/hole / e $Å^{-3}$	0.52/-0.48

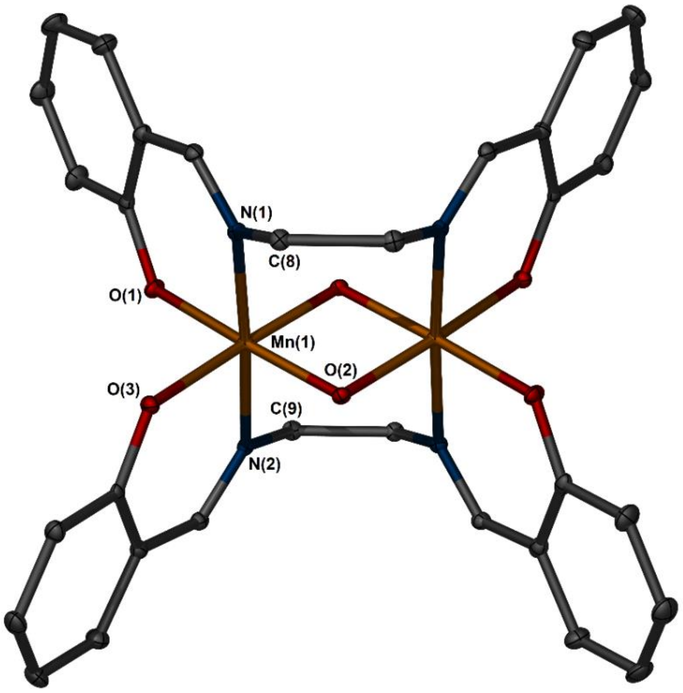

Figure S30. Molecular structure of complex **7a**.

Crystallographic details for 7e:

Identification code	MRC265
Empirical formula	$C_{52}H_{60}Mn_2N_8O_{10}$
Formula weight	1066.96
Temperature/K	120.01(10)
Crystal system	monoclinic
Space group	C2/c
a/Å	21.6125(3)
b/Å	15.41812(18)
c/Å	16.4514(2)
α/°	90.00
β/°	110.1828(16)
γ/°	90.00
Volume/$Å^3$	5145.37(12)
Z	4
ρ_{calc}g/cm^3	1.377

μ/mm^{-1}	4.535
F(000)	2232.0
Crystal size/mm^3	0.4209 × 0.2562 × 0.097
Radiation	CuKα (λ = 1.54184)
2Θ range for data collection/°	10.1 to 147.4
Index ranges	-13 ≤ h ≤ 25, -18 ≤ k ≤ 19, -20 ≤ l ≤ 15
Reflections collected	10541
Independent reflections	5006 [R_{int} = 0.0485, R_{sigma} = 0.0563]
Data/restraints/parameters	5006/3/325
Goodness-of-fit on F^2	1.035
Final R indexes [I>=2σ (I)]	R_1 = 0.0505, wR_2 = 0.1356
Final R indexes [all data]	R_1 = 0.0579, wR_2 = 0.1440
Largest diff. peak/hole / e Å^{-3}	0.67/-0.39

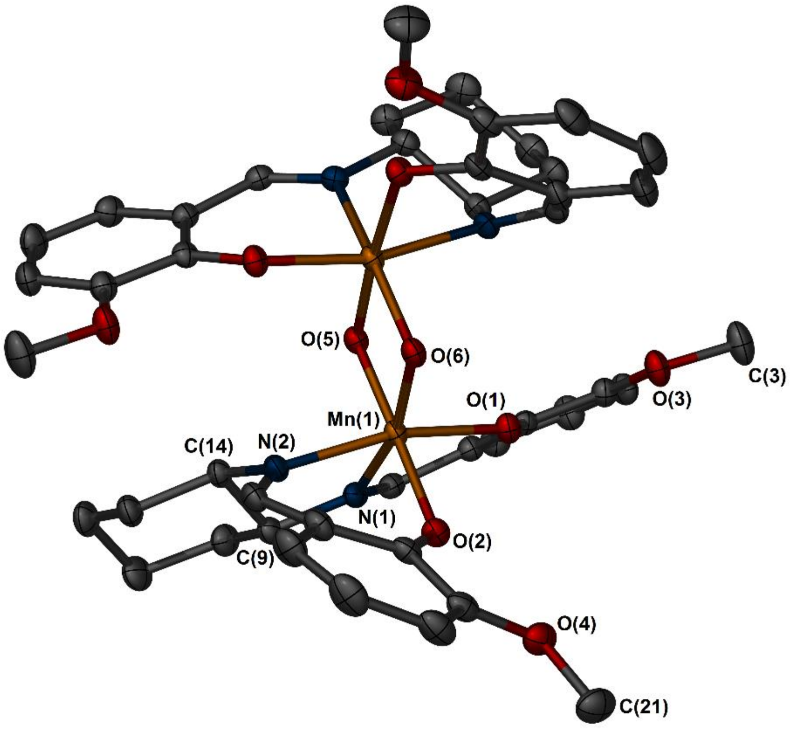

Figure S31. Molecular structure of complex **7e**.

Crystallographic details for 8e:

Identification code	MRC267_Cu
Empirical formula	$C_{24}H_{31}MnN_2O_5S$
Formula weight	514.51
Temperature/K	120.03(10)
Crystal system	triclinic
Space group	P-1
a/Å	9.6974(4)
b/Å	11.4783(4)

c/Å	11.9218(5)
α/°	73.001(4)
β/°	66.826(4)
γ/°	87.881(3)
Volume/Å^3	1162.03(8)
Z	2
ρ_{calc}g/cm^3	1.470
μ/mm^{-1}	5.785
F(000)	540.0
Crystal size/mm^3	0.1473 × 0.0993 × 0.0539
Radiation	CuKα (λ = 1.54184)
2Θ range for data collection/°	8.08 to 147.7
Index ranges	-11 ≤ h ≤ 10, -14 ≤ k ≤ 13, -14 ≤ l ≤ 13
Reflections collected	8887
Independent reflections	4372 [R_{int} = 0.0475, R_{sigma} = 0.0608]
Data/restraints/parameters	4372/0/320
Goodness-of-fit on F^2	1.022
Final R indexes [I>=2σ (I)]	R_1 = 0.0596, wR_2 = 0.1468
Final R indexes [all data]	R_1 = 0.0665, wR_2 = 0.1534
Largest diff. peak/hole / e Å^{-3}	1.35/-1.05

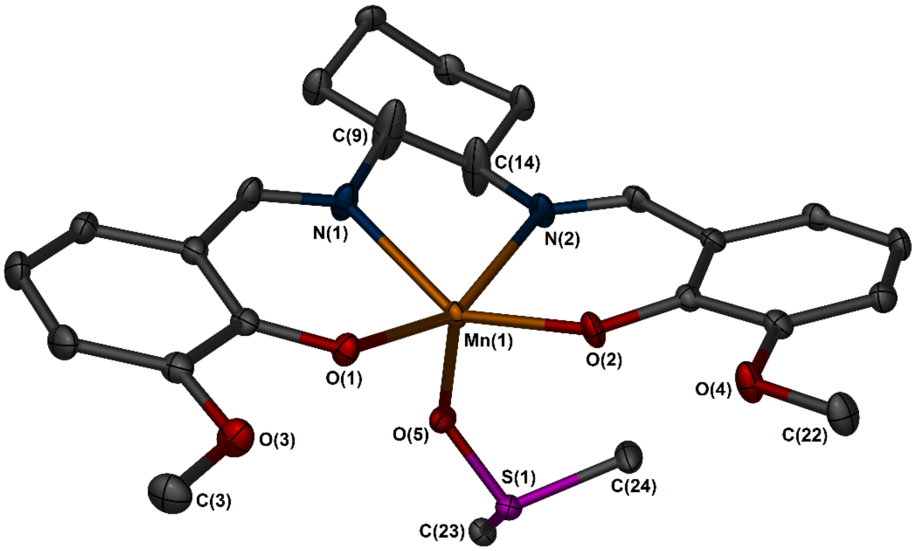

Figure S32. Molecular structure of complex **8e**.

Crystallographic details for 5:

Identification code	MRC223
Empirical formula	$C_{22}H_{23}FeN_2O_4$
Formula weight	435.27
Temperature/K	120.01(15)
Crystal system	monoclinic
Space group	$P2_1/c$
a/Å	10.7886(16)
b/Å	9.8899(12)
c/Å	18.666(3)
α/°	90.00
β/°	101.890(14)
γ/°	90.00
Volume/Å^3	1948.9(5)
Z	4
ρ_{calc}g/cm^3	1.483
μ/mm^{-1}	0.806
F(000)	908.0
Crystal size/mm^3	0.162 × 0.0514 × 0.0299
Radiation	MoKα (λ = 0.71073)
2Θ range for data collection/°	6.36 to 62.5
Index ranges	$-14 \leq h \leq 14$, $-13 \leq k \leq 13$, $-26 \leq l \leq 27$
Reflections collected	13843
Independent reflections	5531 [R_{int} = 0.0794, R_{sigma} = 0.1368]
Data/restraints/parameters	5531/0/263
Goodness-of-fit on F^2	1.095
Final R indexes [I>=2σ (I)]	R_1 = 0.0877, wR_2 = 0.1389
Final R indexes [all data]	R_1 = 0.1512, wR_2 = 0.1605
Largest diff. peak/hole / e Å^{-3}	0.98/-0.51

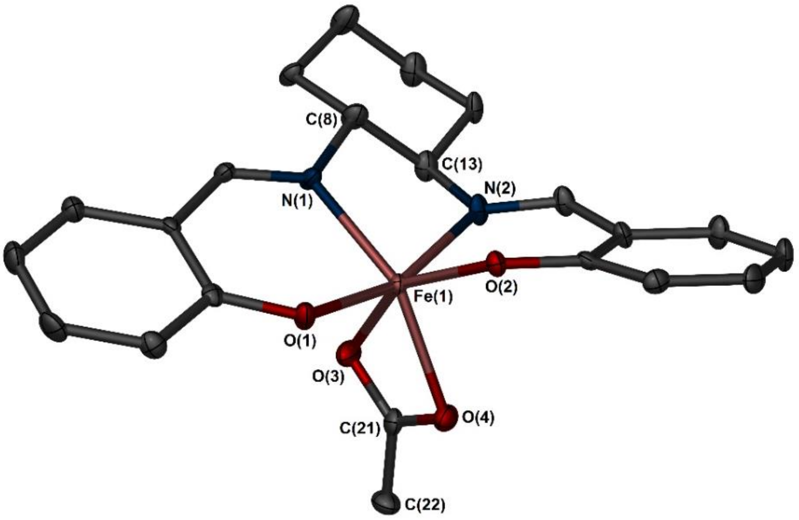

Figure S33. Molecular structure of complex **5**.

Crystallographic details for 7:

Identification code	MRC258
Empirical formula	$C_9H_6Mn_{0.5}NO_2$
Formula weight	187.62
Temperature/K	110.01(10)
Crystal system	orthorhombic
Space group	Pmmn
a/Å	6.5251(2)
b/Å	16.1505(4)
c/Å	7.4488(2)
α/°	90.00
β/°	90.00
γ/°	90.00
Volume/Å^3	784.98(4)
Z	4
ρ_{calc}g/cm^3	1.588
μ/mm^{-1}	7.080
F(000)	382.0
Crystal size/mm^3	0.0918 × 0.0692 × 0.0278
Radiation	CuKα (λ = 1.54184)
2Θ range for data collection/°	10.96 to 147.96
Index ranges	$-7 \leq h \leq 8$, $-7 \leq k \leq 19$, $-8 \leq l \leq 8$
Reflections collected	1936
Independent reflections	859 [R_{int} = 0.0387, R_{sigma} = 0.0374]
Data/restraints/parameters	859/0/79
Goodness-of-fit on F^2	1.191
Final R indexes [I>=2σ (I)]	R_1 = 0.0905, wR_2 = 0.2631
Final R indexes [all data]	R_1 = 0.0911, wR_2 = 0.2634
Largest diff. peak/hole / e Å^{-3}	1.63/-1.22

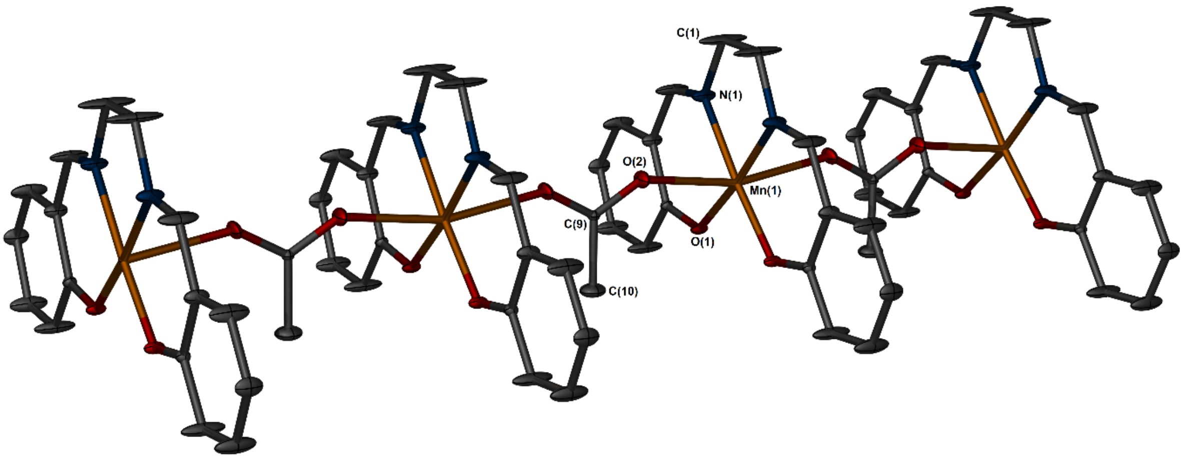

Figure S34. Molecular structure of complex **7**.

19. References

1. F. M. Kerton, S. Holloway, A. Power, R. G. Soper, K. Sheridan, J. M. Lynam, A. C. Whitwood, C. E. Willans, *Can. J. Chem.* **2008**, *86*, 435.
2. D. Nartop, W. Clegg, R. W. Harrington, R. A. Henderson, C. Y. Wills, *Dalton Trans.* **2014**, *43*, 3372.
3. T. A. Immel, M. Grützke, E. Batroff, U. Groth, T. Huhn, *J. Inorg. Biochem.* **2012**, *106*, 68.
4. D. –N. Lee, H. Kim, L. Mui, S. –W. Myung, J. Chin, H. –J. Kim, *J. Org. Chem.* **2009**, *74*, 3330.
5. (*a*) Y. N. Belokon, J. Fuentes, M. North, J. W. Steed, *Tetrahedron* **2004**, *60*, 3191; (*b*) C. G. Hanmaker, G. A. Mirafzal, L. K. Woo, *Organometallics* **2001**, *20*, 5171.
6. S. K. Sur, *J. Magn. Reson.* **1989**, *82*, 169.
7. O. Kahn, *Molecular Magnetism;* VCH Publishers: Weinheim **1993**, 2.
8. O. V. Dolomanov, L. J. Bourhis, R. J. Gildea, J. A. K. Howard, H. Puschmann, *J. Appl. Crystallogr*. **2009**, *42*, 339.
